# The Oldest Case of Decapitation in the New World (Lapa do Santo, East-Central Brazil)

**DOI:** 10.1371/journal.pone.0137456

**Published:** 2015-09-23

**Authors:** André Strauss, Rodrigo Elias Oliveira, Danilo V. Bernardo, Domingo C. Salazar-García, Sahra Talamo, Klervia Jaouen, Mark Hubbe, Sue Black, Caroline Wilkinson, Michael Phillip Richards, Astolfo G. M. Araujo, Renato Kipnis, Walter Alves Neves

**Affiliations:** 1 Department of Human Evolution, Max Planck Institute for Evolutionary Anthropology, Leipzig, Germany; 2 Laboratório de Estudos Evolutivos Humanos, Departamento de Genética e Biologia Evolutiva, Instituto de Biociências, Universidade de São Paulo, São Paulo, Brazil; 3 Instituto de Ciências Humanas e da Informação, Universidade Federal do Rio Grande, Rio Grande, Brazil; 4 Department of Archaeology, University of Cape Town, Rondebosch, South Africa; 5 Departament de Prehistòria i Arqueologia, Universitat de València, València, Spain; 6 The Ohio State University, Department of Anthropology, Columbus, Ohio, United States of America; 7 Instituto de Investigaciones Arqueológicas y Museo, Universidad Católica der Norte, San Pedro de Atacama, Chile; 8 University of Dundee, Centre for Anatomy & Human Identification, Dundee, United Kingdom; 9 Department of Anthropology, University of British Columbia, Vancouver, Canada; 10 Laboratório Interdisciplinar de Pesquisas em Evolução, Cultura e Meio Ambiente, Museu de Arqueologia e Etnologia, Universidade de São Paulo, São Paulo, Brazil; 11 Scientia Consultoria Científica Ltda., São Paulo, Brazil; New York State Museum, UNITED STATES

## Abstract

We present here evidence for an early Holocene case of decapitation in the New World (Burial 26), found in the rock shelter of Lapa do Santo in 2007. Lapa do Santo is an archaeological site located in the Lagoa Santa karst in east-central Brazil with evidence of human occupation dating as far back as 11.7–12.7 cal kyBP (95.4% interval). An ultra-filtered AMS age determination on a fragment of the sphenoid provided an age range of 9.1–9.4 cal kyBP (95.4% interval) for Burial 26. The interment was composed of an articulated cranium, mandible and first six cervical vertebrae. Cut marks with a v-shaped profile were observed in the mandible and sixth cervical vertebra. The right hand was amputated and laid over the left side of the face with distal phalanges pointing to the chin and the left hand was amputated and laid over the right side of the face with distal phalanges pointing to the forehead. Strontium analysis comparing Burial 26’s isotopic signature to other specimens from Lapa do Santo suggests this was a local member of the group. Therefore, we suggest a ritualized decapitation instead of trophy-taking, testifying for the sophistication of mortuary rituals among hunter-gatherers in the Americas during the early Archaic period. In the apparent absence of wealth goods or elaborated architecture, Lapa do Santo’s inhabitants seemed to use the human body to express their cosmological principles regarding death.

## Introduction

Few Amerindian habits impressed the European colonizers more than the taking and displaying of human body parts, especially when decapitation was involved [1]. Although disputed by some authors [2], it has become widely accepted that decapitation was common among Native Americans across the entire continent and the archaeological evidence confirms that the practice has deep chronological roots [3]. In South America, the oldest decapitation is reported for the Andean region and dates to ca. 3000 BP at the site of Asia 1, Peru. Since all other South American archaeological cases occur in the Andes (e.g., Nazca, Moche, Wari, Tiwanaco) it was assumed that decapitation was an Andean phenomenon in both its origins and in its most unambiguous expression. In the present contribution we review the available evidence on decapitation in South America and report the discovery in east-central Brazil of a case of human decapitation directly dated to 9127–9438 cal BP (all chronological ranges reported here are based on a 95.4% interval). Excavated at the Lapa do Santo rock shelter in Lagoa Santa, Central Brazil, this is the oldest case of decapitation found in the New World, leading to a re-evaluation of the previous interpretations of this practice, particularly with regards to its origins and geographic dispersion.

### Disembodied heads and decapitation in South America

In South America, the practice of decapitation is reported in both the ethnographic and archaeological literature. Tupinamba groups from coastal Brazil, famous for their rituals, including exo-cannibalism [4], used to collect body parts, including heads, as war trophies [5]. The Arara Indians, in the Brazilian Amazon, performed the Ieipari ceremony in which the cranium of the defeated enemy, also used as a musical instrument, was displayed on the top of a pole [6]. Among the Uru-Uru Chipayas, in Bolivia, skulls were used as part of a syncretic Christian liturgy [7]. Among the Inca, decapitation was a common means of establishing and reinforcing positions of status and power. The head of important enemies were turned into trophies and the skulls into drinking jars in a clear message of military supremacy [8]. However, among the ethnographic examples in which decapitation was prominent, the trophy heads made by the Munduruku and Jivaros are the most famous.

The Munduruku Indians from the Tapajós River in northern Brazil used to behead the defeated enemy immediately after death [9–16]. The spine was sectioned near the foramen magnum and the head removed. The internal muscles, brain, eyes and tongue were then removed [16] and the head mummified through immersion in hot oil and subsequent smoking [15]. The trophy would be brought to the village and designated as the focus of a series of ceremonies over several years. At first, the ritual involved the cultural appropriation of the trophy by adding ornaments and tattoos to it. Subsequently, as the power of the head faded away, the skin and the ornaments were removed. Finally, the dentition was extracted from the skull and attached to a cotton belt that would remain with the owner of the head indefinitely, while the skull itself would be left in some corner of his habitation to be forgotten [13].

For the Munduruku, the head of the defeated enemies clearly served the role of a war trophy and symbol of belligerent superiority [14,16]. The head was sometimes positioned on the end of a long pole [11] or carried by strings attached to the cranium, clearly characterizing the importance of public display [13]. At the same time, the head was an empowering object capable of increasing success in hunting and incorporating a female semiology of fertility. Although the Munduruku would remove other body parts of their own dead, they only produced trophy heads with enemies. The enemy’s children were commonly captured and incorporated into the community but never used to generate trophy heads [15].

In Ecuador, the Jivaros produced shrunken heads (*tsantsa*) from dead enemies. The head was quickly removed from the body with a “v-shaped” incision made above the clavicles. Later, in a safer location, the skin of the head was removed from the skull. This scalp was then washed with boiling water for 15–30 minutes resulting in a 50% reduction of the head’s dimensions. The shrunken head was equipped with cords to facilitate transport and handling [17]. Jivaro’s *tsantsa* had the power to imprison the soul of the dead enemy precluding it from perpetrating any vengeance [18–21] (but see Fausto and Rodgers (1999) [22] for a broader perspective on the meaning of *tsantsa*).

Some authors suggested that the practices of head-hunting were not a truly indigenous phenomena but a result of the western commercial demands for trophy heads [2]. However, although the European market certainly catalyzed the practice of head-hunting in South America, leading to a transformation of the reasoning behind it, archaeological evidence confirms that similar practices were common long before the arrival of the European colonizers [23,24].

The Chimus (900AD-1470AD) in Peru incorporated decapitation as a standard procedure in human sacrifices. In the Huaca 1 Complex of Pacatnamu, the mutilated skeletons of 14 individuals were found within a defensive trench of three meters deep. The ubiquitous presence of young males, many of which were tied and left exposed after death, suggests that these were sacrificed defeated warriors. Among the diverse types of mutilation to which they were subjected, decapitation was one of them [25]. Chimu human sacrifices also took place in the Temple of the Sacred Stone in Tucume [26]. Osteological analysis suggests a ritual sequence starting with throat cutting followed by heart extraction and ending with decapitation (a total of 72 individuals presented explicit osteological evidence of decapitation). The severed heads were buried in the same pit with the correspondent headless body. The presence of children among the sacrificed individuals makes it unlikely that these were defeated warriors, pointing to a different sort of sacrificial ritual compared to Pacatnamu. Disembodied skulls of both adults and children were also used as dedicatory offerings and were included in tombs as individualized objects wrapped in textile accompanying the remains of sacrificed individuals [27].

Among the Chachapoyas from the Peruvian Amazon, disembodied skulls are found on top of elaborated anthropomorphic sarcophagi used as funerary monuments (e.g., Karajia) [28]. Disembodied skulls were also found in the walled city of Kuelap. In either case, detailed osteological analyses are not available, and the interpretations about the disembodied skulls range from them being considered simply delayed burials to being war trophies [29,30].

In the Wari Empire (600AD-1100AD), in southern Peru, disembodied heads were transformed into trophies and played a central role in ritualistic traditions [31]. In the site of Conchopata at least 31 trophy heads were recovered from ritual structures (EA143 and EA72) [32]. The skulls show drill holes near the bregma and, sometimes, at the occipital bone [7]. The demographic profile of Wari’s trophy heads shows a predominance of male individuals of all ages, including children [32]. Isotopic analyses suggest a non-local origin for some of the decapitated individuals and osteological evidence points to high levels of inter-personal violence [33]. Altogether, and including the practice of child abduction, decapitation in Wari is understood as a strategy adopted by military and ritual elites to legitimate their authority in the eyes of their enemies. However, not all disembodied skulls found in Wari contexts were trophy heads. In the site of Wari, a non-modified skull wrapped in cloth and pinned with four copper *tupus* was found under the floor of an architectural construction and was probably a dedicatory offering [34].

During the Tiwanaku period (300AD-1000AD), in the Titicaca basin in Bolivia, scenes involving decapitation or disembodied human heads were a common theme in the etchings of their rock sculptures and panels [35]. The osteoarchaeological record for the corresponding period confirms that these were indeed a real practice. In the high-status residential complex of Putuni (west to the Kalasasaya) a total of fifteen articulated and disarticulated individuals were buried as a dedicatory offering to the building, including a disembodied human skull [36,37]. In the pyramid of Akapana, a site of communal ritual in the core of the Tiwanaku complex, isolated human bones or partially articulated skeletons were recovered from the base of some of the excavated pyramid’s walls. Several skulls were found isolated (in one case, three skulls were grouped together), and eighteen skeletons lacked their skulls [35]. In the absence of cut marks, the skulls must have been removed from the skeleton in secondary contexts, which has been suggested to be a part of an “esoteric cult of the head” [36]. In the site of Wata Wata, human heads were presented as dedicatory offerings [38]. Three disembodied skulls were found displaying different signs of *perimortem* violence, including beheading, cranial and facial fracturing, defleshing, jaw removal, and possible eye extraction. The extreme violence characterizing these findings suggests this was done to remove power from those individuals and legitimize the authority of the expanding influence of Tiwanaku into the region [38].

Head removal is a common theme in Moche (100AD-700AD) iconography, in northern Peru [39–42], and archaeological and osteological evidence abound to confirm this was not merely figurative but a real practice. In Plaza 3A and Plaza 3C of Huaca de la Luna [34,43–50], articulated severed heads and decapitated bodies were found in a context of generalized sacrifice of defeated warriors [49,51,52]. In Plaza 3C, in addition to the ritual of sacrifice, the severed skulls were also subject to both *peri* and *postmortem* intentional manipulation which could imply some sort of ritual cannibalism [49]. Nearby, at the complex named ZUM 8, two disembodied skulls altered to function as jars show the diversity of purposes head removal had among the Moche, going beyond the immediate needs of sacrificing defeated warriors [45,48]. In Huaca Dos Cabezas, a cache of 18 severed skulls with cut marks on the anterior portion of the cervical vertebrae was found [41]. Nearby, the complete skeleton of a tall man was found with a *tumi* (ceremonial axe characterized by a semi-circular blade) in his left hand and a pottery human head in his right hand, suggesting he was an actual decapitator. In San José del Moro (tomb M-U1221), seven individuals were buried together and eight disembodied skulls were placed on top of the burial [53]. The presence of several pottery artifacts related to shamanistic activities [54] suggests that the skulls are grave offerings, possibly holding some supernatural power. During the Moche period, human bones from reopened tombs were used as dedicatory offerings. Skulls were the most commonly selected anatomical part and therefore not all disembodied heads or headless bodies are a product of decapitation (i.e., *perimortem* removal of the head) [55,56]. In addition to humans, llamas’ decapitated heads were also included in tombs and graves (e.g., Huaca Rajada Sipán [57] and Dos Cabezas Tomb 2 [58,59]). During the earlier Gallinazo period, in Huacas de Moche, a single case of skull removal is known for burial G2. The skull was removed and replaced by a pottery jar with the figure of a human head stamped on it. It is not possible, however, to determine if this was a *peri* or *postmortem* removal [60].

The Nazca (100BC-800AD), in southern coastal Peru, produced elaborate trophy heads that were characterized by a drill hole in the front of the head and an enlargement of the foramen magnum [61–67]. The lips and eyes were usually sealed with spines and the head was equipped with a carrying string [34]. The available iconography and the predominance of adult males among trophy heads [34] indicates that decapitation took place in the battlefield, and that the severed head functioned as a trophy of war. Isotopic analysis indicates that these were intra-valley battles involving local Nazca warriors [68,69]. The heads were commonly interred in caches in numbers ranging from three to groups of 40 or more [66,70]. Therefore, their significance went far beyond signaling military supremacy, and it is assumed they were a central element in rituals aiming to control the forces of nature, particularly concerning crop fertility [64,71–73].

In the site of Chavín de Huantar (1200BC-500BC), in the northern Peruvian highlands, four disembodied skulls were found on a platform (Urabarriu phase, 900BC-500BC). Since the skulls were from an old adult male, a young adult male, an adolescent female and an infant, they are sometimes thought to represent an extended family [74]. The skulls show no signs of modification. Another isolated skull in Chavin de Huantar was recovered from the Galeria de Ofrendas and, although a precise date is not available, this could represent the earliest modified trophy head in the Andes [31,75].

During the Formative period, five disembodied skulls were found in the site of Wichquana, in Peru. Buried in individual pits within a ceremonial structure these skulls still had the cervical vertebrae articulated to them supporting the interpretation that they were decapitated when soft tissue was still present, which suggests that they were sacrificed [76]. The site of Asia 1 [77], in central coastal Peru, is usually considered the oldest possible case of decapitation in South America (ca. 3000 BP) [31,45]. However, in the absence of a detailed osteological description accounting for the presence of cut marks in the cranium and associated cervical vertebrae, it is not possible to determine if this in indeed a case of decapitation. The findings consisted of three wrapped bundles containing a total of eight disembodied heads that were found in separate graves. In addition, two headless bodies were also present. One skull had cut marks on the frontal bone that were interpreted as resulting from the scalping of the face [77]. The funerary context included several textiles, a necklace of bone disks, shell pendants, a bone pin, feathers, red pigment and an “engraved tray holding a mirror” [77]. Such an elaborate treatment indicates that the practice of removing skulls in Asia 1 could have been reserved to individuals of special status. Altogether, and considering the lack of any further modification to the skulls, it seems they were less likely trophy heads, but instead venerated members of this society. Accordingly, it has been suggested that the flayed skull might represent a local individual who was mutilated somewhere else and later brought back to Asia 1 [34].

The site of Asia 1 is commonly mentioned as the first appearance of disembodied heads in the South American archaeological record. However, Aguazuque (5025–2725 BP) might be a better candidate. Located in Sabana de Bogotá, Colombia, at least two cases of disembodied skulls and one headless body were identified among a total of 59 burials. The site presents one of the most elaborate funerary records of the Archaic period and the disembodiment of the skulls were part of a broader mortuary context that was focused on the manipulation of bones and body parts [78–80]. Long bones, for example, were sectioned into diaphyses and epiphyses and further painted with geometric motifs. Once again, in the absence of a detailed osteological description accounting for the presence or absence of cut marks, it is not possible to determine if these were true cases of decapitations. Notwithstanding, the fact that one of the disembodied skulls was articulated with the cervical vertebrae is highly suggestive that the removal occurred while soft tissue was still present and therefore characterizes a case of decapitation.

In Brazil, as far as we could determine, there is only one single case of a possible decapitation reported for the entire pre-history of the country. This finding comes from the shellmound of Forte Marechal Luz [81], but no detailed chronology or osteological descriptions are available. Therefore, it is clear that almost all reported archaeological cases of decapitation and disembodied heads in South America are concentrated in the Andean region [82]. For this reason it is commonly assumed that this was an Andean phenomenon in both its origins and in its most unambiguous expression [2,24,40,72]. The purpose of the present publication is to contribute to the field by reporting an early Holocene case of decapitation found in Lagoa Santa, east-central Brazil.

### The Lagoa Santa region

**Fig 1:**
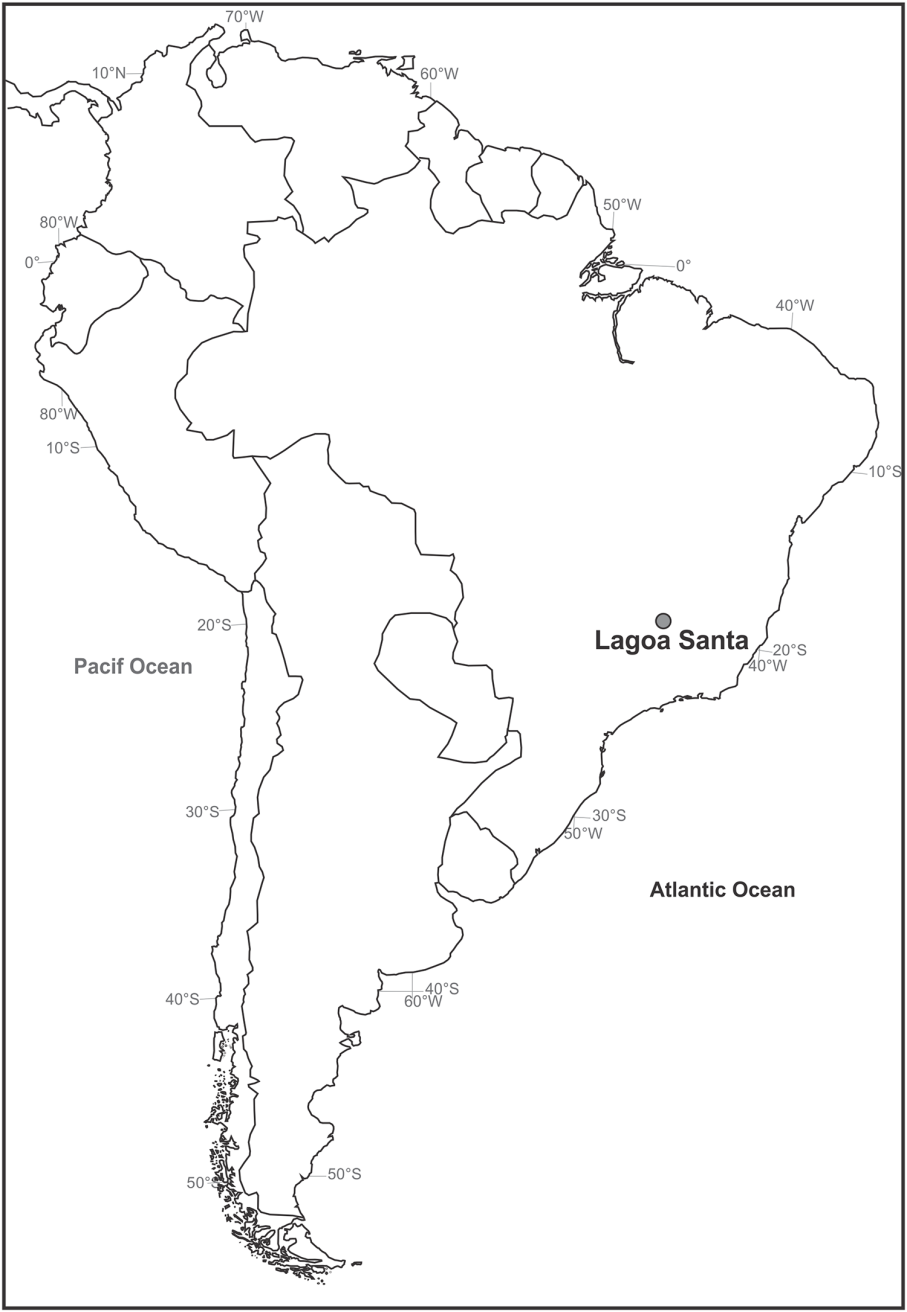
Map of South America. The location of Lagoa Santa is indicated by the dot.

Lagoa Santa is an environmentally protected area comprising 360 km^2^ located in east-central Brazil (Fig 1). The vegetation is dominated by *cerrado* (a savannah-like vegetation) and semi-deciduous forest. The rivers Mocambo, Samambaia, Jaguara and Gordura make up a tributary net that flows west to east towards the Velhas River, the main river in the area. Geomorphologically, Lagoa Santa is a karstic terrain that can be divided into four distinct domains [83]: 1) below 660 meters above sea level (masl), the terrain is characterized by a fluvial plain connected with the regional base level (Velhas River); 2) between 660 and 750 masl, there is a karstic plain with dolines and lakes 3) between 750 and 850 masl, there are karstic plateaus characterized by the presence of limestone outcrops (reaching up to 75 meters in height); 4) above 850 masl, residual peaks composed of the non-soluble meta-sedimentary rocks from the Serra da Santa Helena Formation.

The region’s geology comprises the Sete Lagoas Formation and the Serra da Santa Helena Formation, both part of the Upper Proterozoic meta-sediments of the Bambuí Group [84] of the São Francisco craton. This cratonic cover metamorphosed during the Brazilian Cycle (700–450 million years ago) in a process that resulted in planar structures, such as lineation and foliation, and sub-vertical structures, such as normal and revert faults. The combination of these structures provides the path for the geomorphologic evolution that leads to the rock shelter configurations found in the region. The regional rock shelters and outcrops are developed in the limestone of the Sete Lagoas Formation. More specifically, Lapa do Santo rock shelter developed in the Member Pedro Leopoldo that is composed of very pure limestone with more than 90% calcite [84].

The annual mean temperature is 23°C, with lower temperatures (11°C) occurring between June and July and higher temperatures (35°C) occurring between October and November. The mean humidity is around 65% in the dry season, from May to September, and around 85% in the rainy season, from November to April, with a pluviometric mean of 1,400 mm/year. The major climatic characteristic of this region is the high concentration of rain during the rainy season (93% of total volume). When evaporation is analyzed, the region presents an annual deficit of 176 mm [85]. Despite these particular variations, the regional climate is classified as tropical, with a rainy summer and a dry winter [86]. During the dry period, the above ground water sources can become very scarce, although underground drainages are capable of preserving the discharge in the Velhas River.

The first prehistoric human bones in Lagoa Santa were found by the Danish naturalist Peter Lund between 1835 and 1844 [87–91]. Due to the putative coexistence of humans and megafauna, Lagoa Santa became a well-known region for 19^th^ century scholars [92–95]. During the 20^th^ century different teams went to the region to find evidence that could confirm the coexistence hypothesis [96–100]. As a result of more than 170 years of excavations, a large collection of early Holocene skeletons was gathered [101–103]. However, all those excavations were done without proper documentation and therefore they lack detailed contextual information. Coordinated by WAN and funded by the São Paulo State Grant Foundation (FAPESP), the project “Origins and Microevolution of Man in America: a Paleoanthropological Approach” aimed to overcome this problem by identifying and excavating new sites in the Lagoa Santa region. Lapa do Santo was excavated within the midst of these efforts.

### Lapa do Santo archaeological record

**Fig 2:**
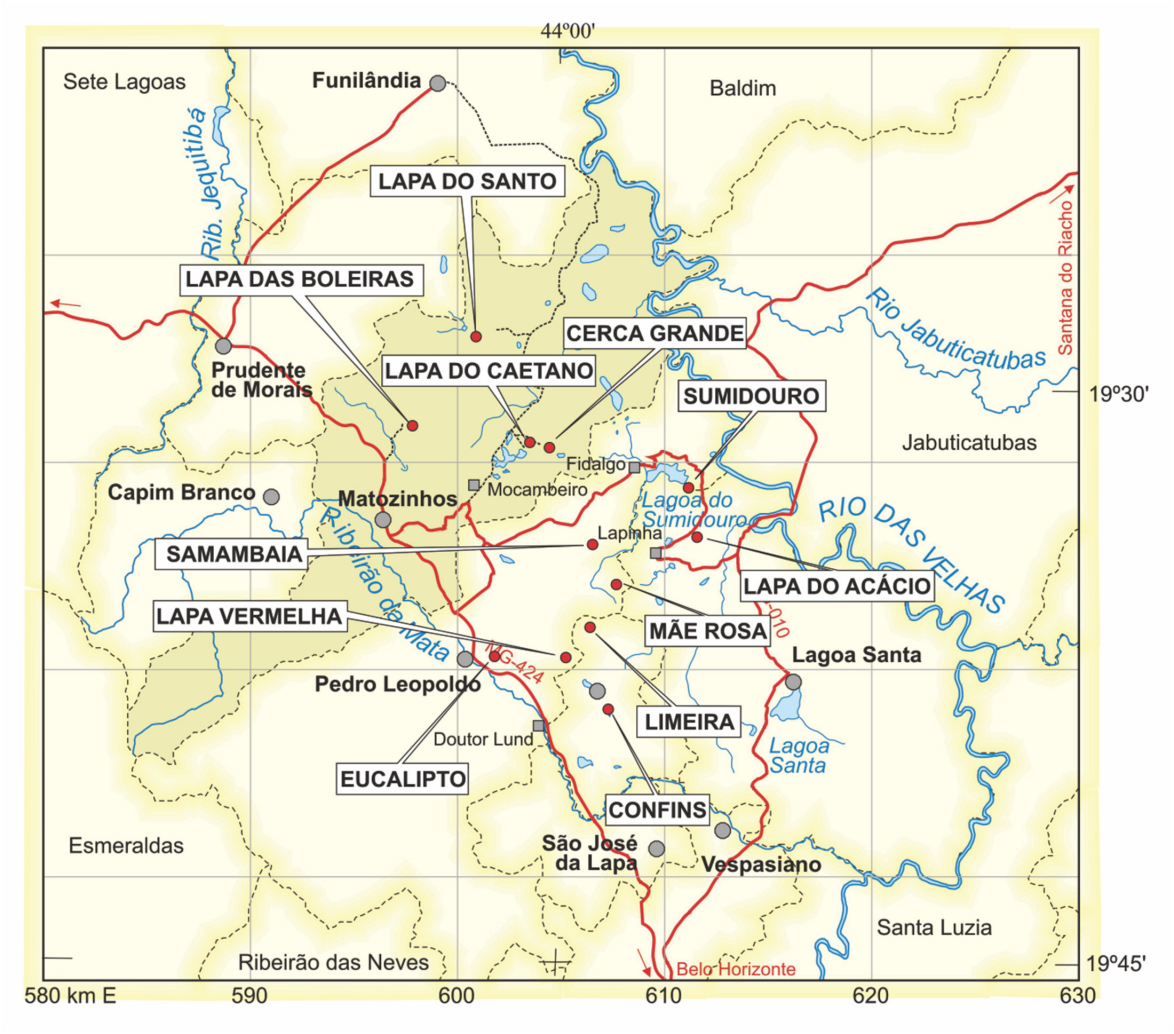
Map of the Lagoa Santa region. The dots indicate all early Holocene sites where human skeletal remains were found.

**Fig 3:**
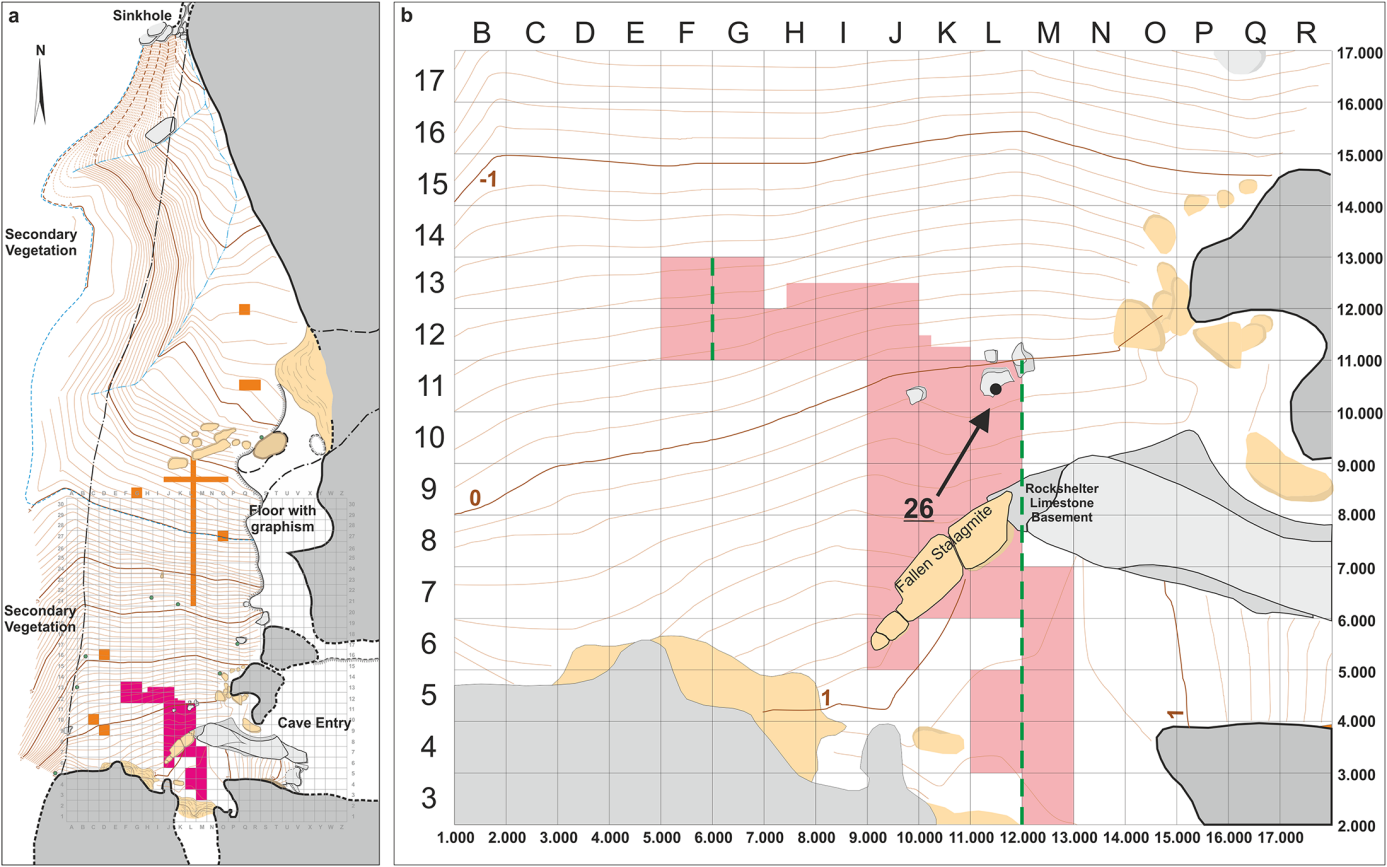
Plan of Lapa do Santo. a) The grid corresponds to 1 square meter units. Purple and orange areas indicate excavated surfaces. Pink area indicates the main excavation area (MEA). The bedrock is depicted in gray, and secondary deposits such as breccia and stalagmites in beige. The topographic lines are 10 cm equidistant and the associated values correspond to the z-value of the site coordinate system. b) Detail of the MEA area. Black disk and the black arrow indicate the position of Burial 26. Numbers in the lower and right margin indicate the x and y values, respectively, from the coordinate system of site.

**Fig 4:**
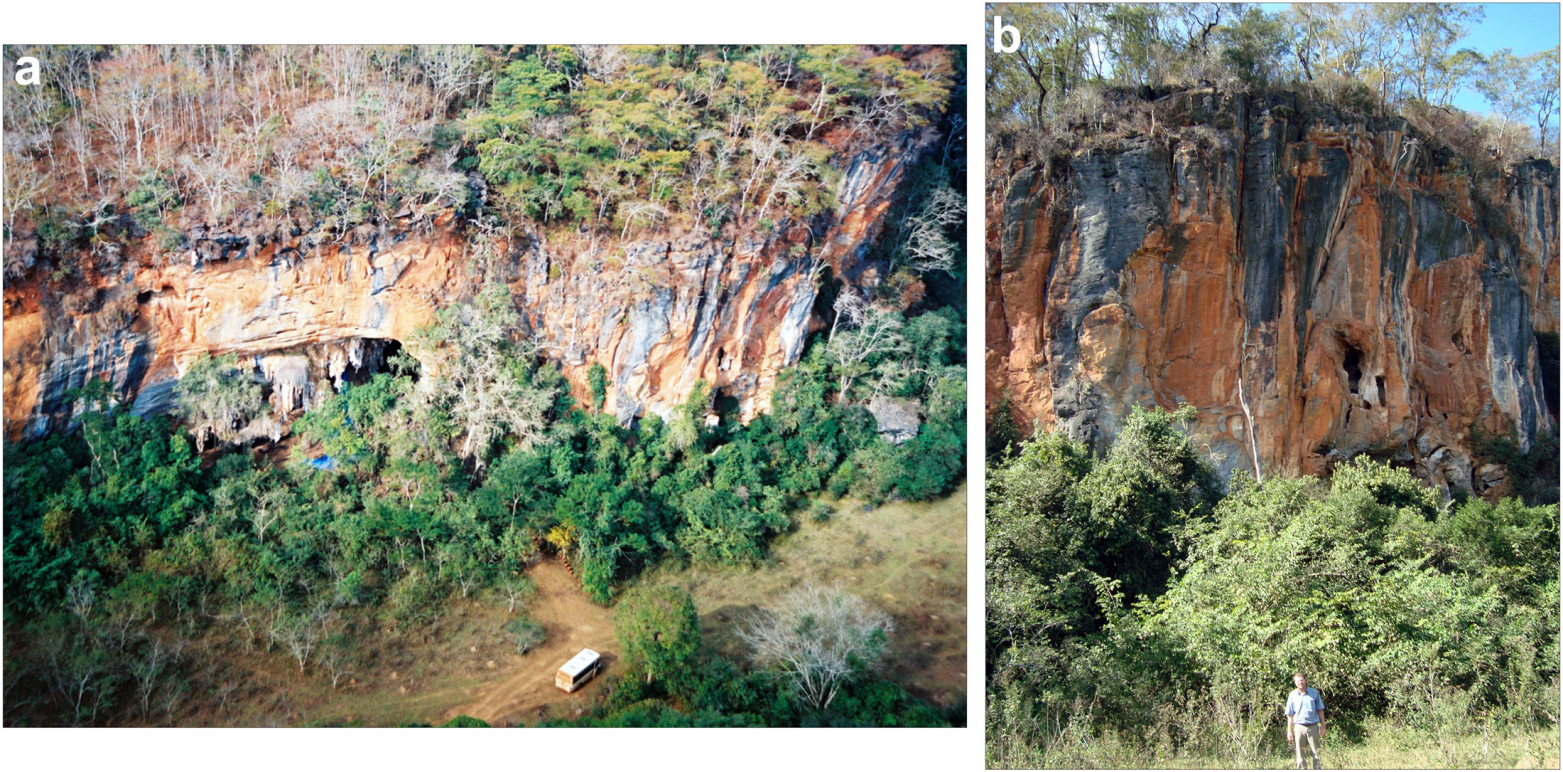
Lapa do Santo massif. a) Aerial view of the massif in which the rock shelter is located; b) ground view of the massif, the site is located just behind the vegetation. The individual in panel 4b has given written informed consent (as outlined in PLOS consent form) to publish this image.

Lapa do Santo (“Saint’s rock shelter”) is an archaeological site located in the northern part of the Lagoa Santa karst (city of Matozinhos, state of Minas Gerais, Brazil, coordinates of the site 19°28'37.86"S and 44°2'17.00"W) (Fig 2) [104]. The site has an associated sheltered area of ca. 1300 m^2^ (Fig 3a) developed under the negative slope of a 30-meter high limestone massif (Fig 4). The southern region of the sheltered area has a relatively flat, high and dry area located immediately in front of the cave’s entrance. The floor of the shelter has a strong descending inclination towards the north, which becomes flat again near a natural sinkhole located in the northern extreme of the sheltered area.

Excavations took place between 2001 and 2009 under the coordination of RK, AGMA and DVB. Starting in 2001 several units were opened in distinct areas of the shelter, which showed that the richest archaeological deposits were located in its southern part, immediately in front of the cave`s entrance. An ample excavation surface was established in this region, becoming the Main Excavation Area (MEA, the highlighted area in Fig 3b). Excavations ended in 2009 when, in accordance to Brazilian laws, the excavated area was filled with sediments to reconstitute the original topography of the shelter`s floor. In 2011 a new excavation area was opened as part of a new research project (“The Mortuary Rituals of the First Americans”), coordinated by AS, and a joint venture between the Department of Human Evolution of the Max Planck Institute for Evolutionary Anthropology (Germany) and the Laboratório de Estudos Evolutivos Humanos da Universidade de São Paulo (Brazil).

The chronology of the site is based on OSL and radiocarbon dates and points to the human presence starting at 12.7–11.7 cal kyBP (95.4% interval). Three distinct periods of occupation were determined based on the radiocarbon dates. Lapa do Santo’s Period 1 (LSP-1) starts at 12.7 cal kyBP and ends at 7.9 cal kyBP; Lapa do Santo’s Period 2 (LSP-2) starts at 5.4 cal kyBP and ends at 3.9 cal kyBP; Lapa do Santo’s Period 3 (LSP-3) starts at 2.1 cal kyBP and ends at 0.0 cal kyBP (see [105] for a detailed account on the site chronology).

Lithic technology [106,107], zooarchaeology [108], and multi-isotopic analyses [109] indicate typical early Archaic groups of hunter-gathers with low mobility and a subsistence strategy focused on gathering plant foods and hunting small and mid-sized mammals [104]. Together with reported frequencies of dental caries comparable to those observed among agricultural populations [103,110,111], the emerging picture for Lagoa Santa during early Holocene is an economy structured around staple carbohydrates complemented by hunting of small and mid-sized animals. Formation process analysis characterizes the Lapa do Santo’s deposits as mainly anthropogenic and composed of repeated combustion activities, indicating an intense occupation of the same locality. The oldest evidence of rock art in South America, including a pictorial tradition that depicts phallic imagery, was also found engraved on the bedrock of Lapa do Santo, under four meters of excavated sediments [112].

A total of 26 human burials dating to early Holocene (LSP-1) were exhumed from Lapa do Santo between 2001 and 2009 (see [105] for a comprehensive depiction of the mortuary practices in Lapa do Santo and the Lagoa Santa region). The use of Lapa do Santo as an interment ground started between 10.3–10.6 cal kyBP. Lapa do Santo Mortuary Pattern 1 (LSMP-1) was characterized by articulated skeletons in flexed position buried in shallow graves and covered by limestone blocks and occurred between 9.7–10.6 cal kyBP. Lapa do Santo Mortuary Pattern 2 (LSMP-2) took place between 9.4–9.6 cal kyBP and was characterized by an emphasis on the reduction of the body by means of mutilation, defleshing, tooth removal and exposure to fire followed by the secondary burial of the remains according to specific rules. The case of decapitation reported here is part of LSMP-2. In the absence of monumental architecture or grave goods, during this period the local groups elaborated their funerary rituals through the use of the human body as a symbol [113]. Lapa do Santo Mortuary Pattern 3 (LSMP-3) took place between 8.2–8.6 cal kyBP when another change occurred whereby pits were instead filled with disarticulated bones of a single individual without signs of body manipulation. In some cases the long bones were highly comminuted in order to fit the small pit.

### The decapitation of Lapa do Santo’s Burial 26

**Fig 5:**
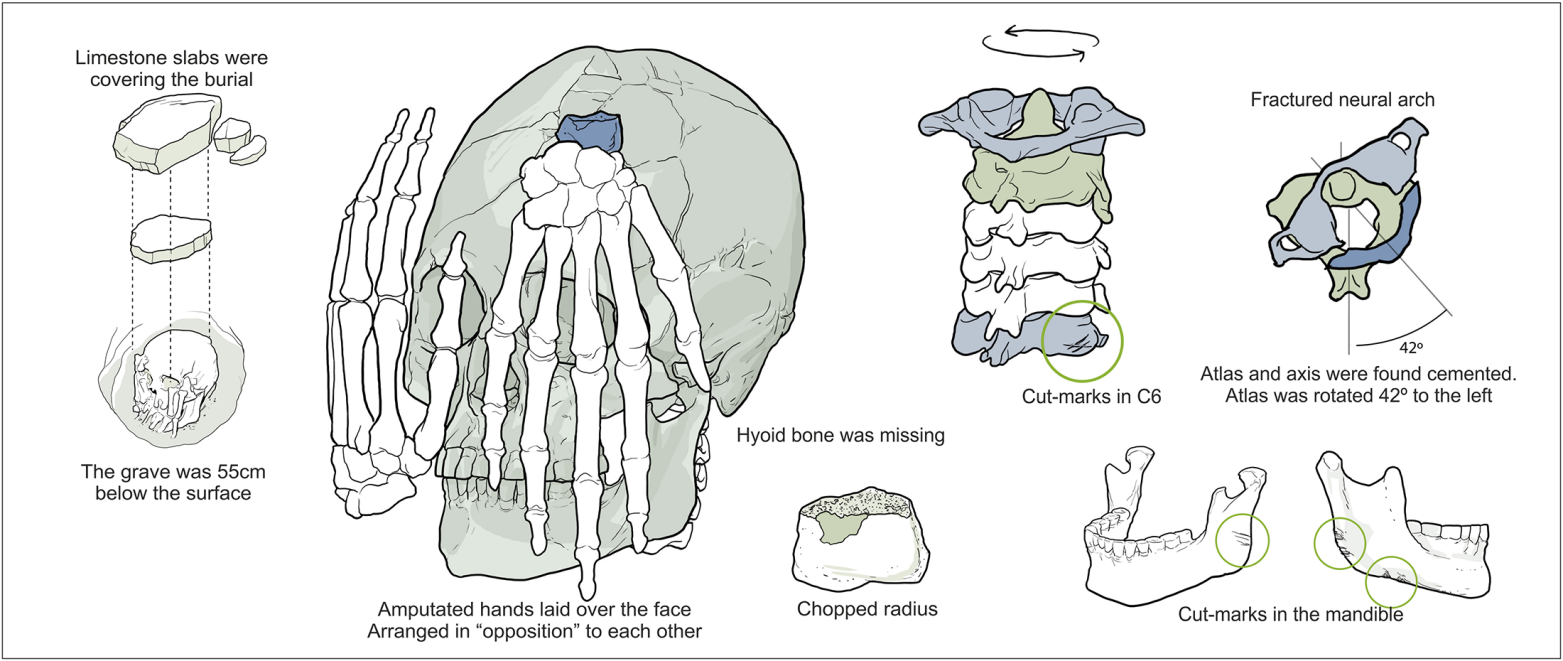
Schematic representation of Burial 26 from Lapa do Santo. Drawing by Gil Tokyo.

The decapitation case that is the focus of the present contribution (accession ID Burial 26, Fig 5) was exhumed from Lapa do Santo in July 2007. The site was excavated under the authorization of the Instituto do Patrimônio Histórico e Artístico Nacional (IPHAN processes: 01514.000329/2000-51, 01516.000236/2005-11, 01514.002967/2011-97) and of the Instituto Chico Mendes de Conservação da Biodiversidade (ICMBio processes: 29395–2 and 29395–3). Burial 26 is today housed in the Laboratory for Human Evolutionary Studies (Department of Genetics and Evolutionary Biology, Instituto de Biociências, Universidade de São Paulo). Permission to study the specimen was granted by the curator of the collection (WAN).

Burial 26 was found on level 10 of unit L11 at 55 cm below the surface (Figs 3a and 7). This area of the site was extensively used for interments and several pits surrounded the grave of Burial 26 but without intercepting it. Burial 26 was composed of three distinct groups of fully articulated bones found as a single interment. The first group comprised the skull with its mandible in occlusion and the first six cervical vertebrae (C1-C6) (Fig 7). The hyoid bone was absent. The second group of articulated bones was composed of the bones of the left hand and the third group consisted of all bones of the right hand and the distal extremity of the right radius (Fig 8). The palms of the hands were positioned over the face of the skull. The right hand was laid over the left side of the face with distal phalanges pointing down (i.e., to the chin), while the left hand was laid over the right side of the face with distal phalanges pointing up (i.e. to the forehead). This assemblage was found within a circular grave of ca. 40cm in diameter filled with loose sediment, which was distinct from the remaining matrix of the site. Five limestone cobbles were found above the bones, but still within the grave’s borders. Using cranial morphology and tooth wear (see SI for details), this individual was estimated to be a young adult male.

**Fig 6:**
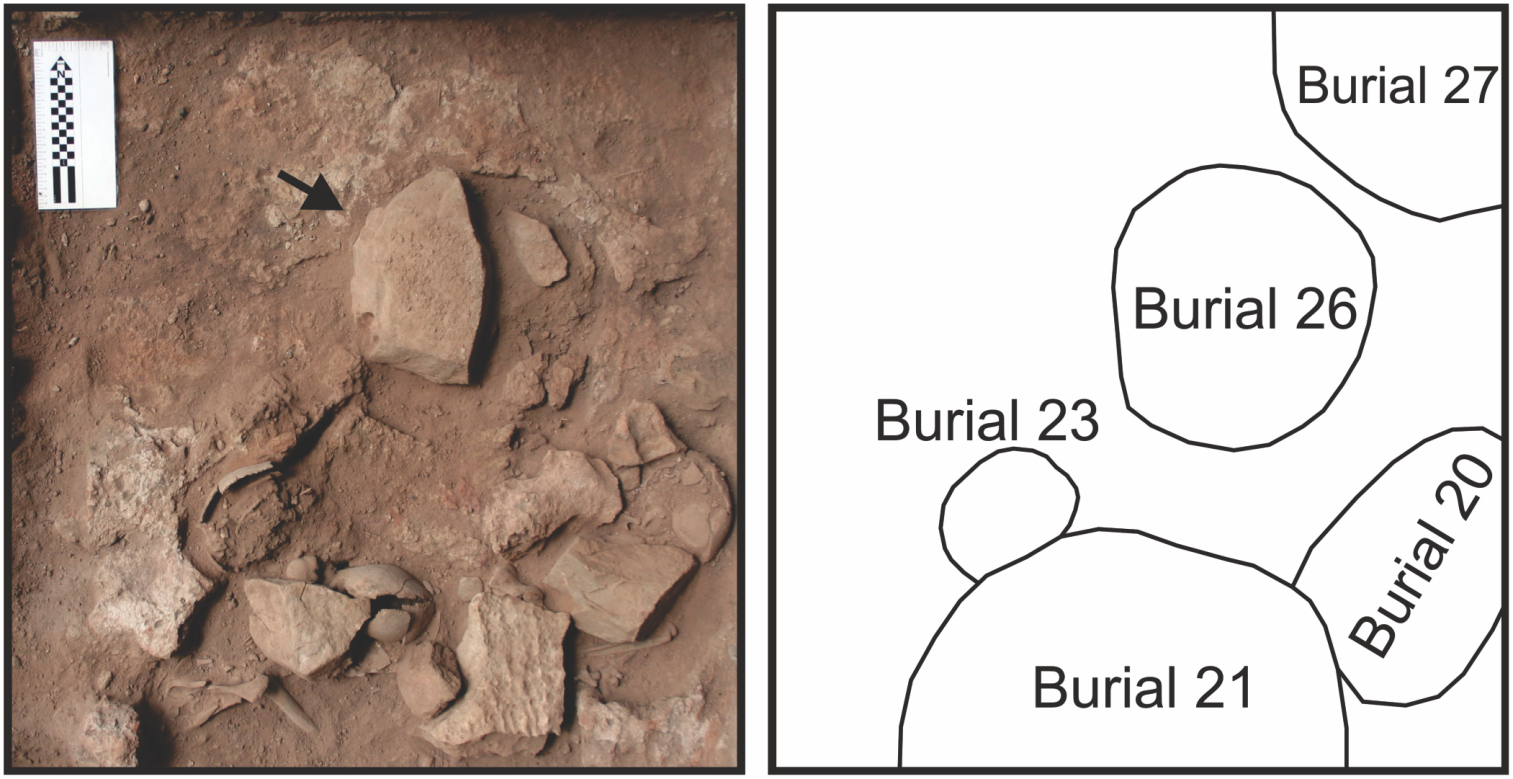
Lapa do Santo unit L11 at level 10. a) Field picture. The black arrow points to the block that marks the upper limit of the pit of Burial 26; b) schematic representation of Unit L11’s level 10, the black contours indicate the approximate limit of each burial.

**Fig 7:**
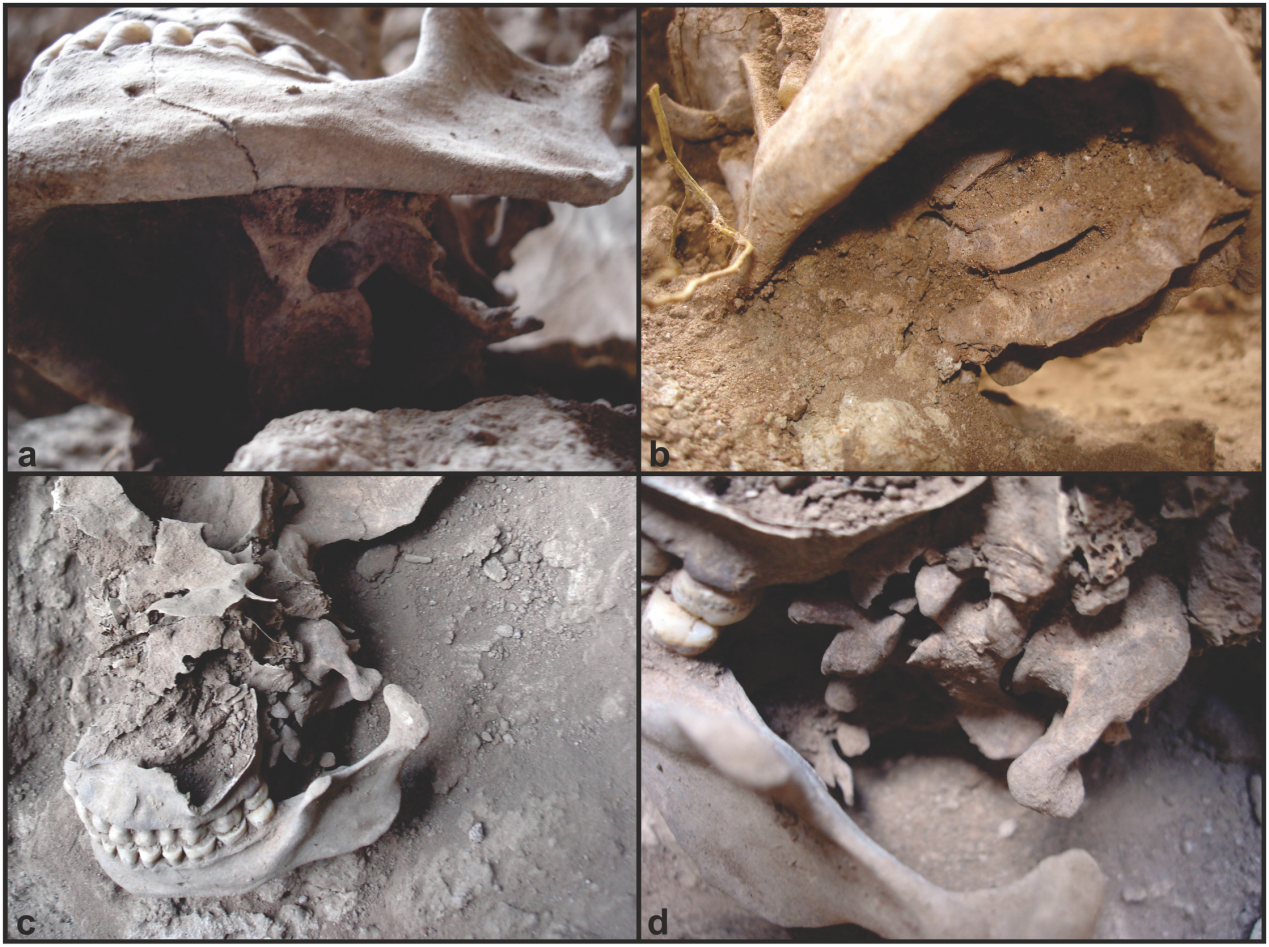
Burial 26. Arrangement of the cervical vertebrae. a) infero-lateral view; b) infero-anterior view; c) the left part of face and neurocranium were removed to allow the view of the relative position of atlas and foramen magnum; d) detail of the relationship of atlas, axis, and the other cervical vertebrae.

**Fig 8:**
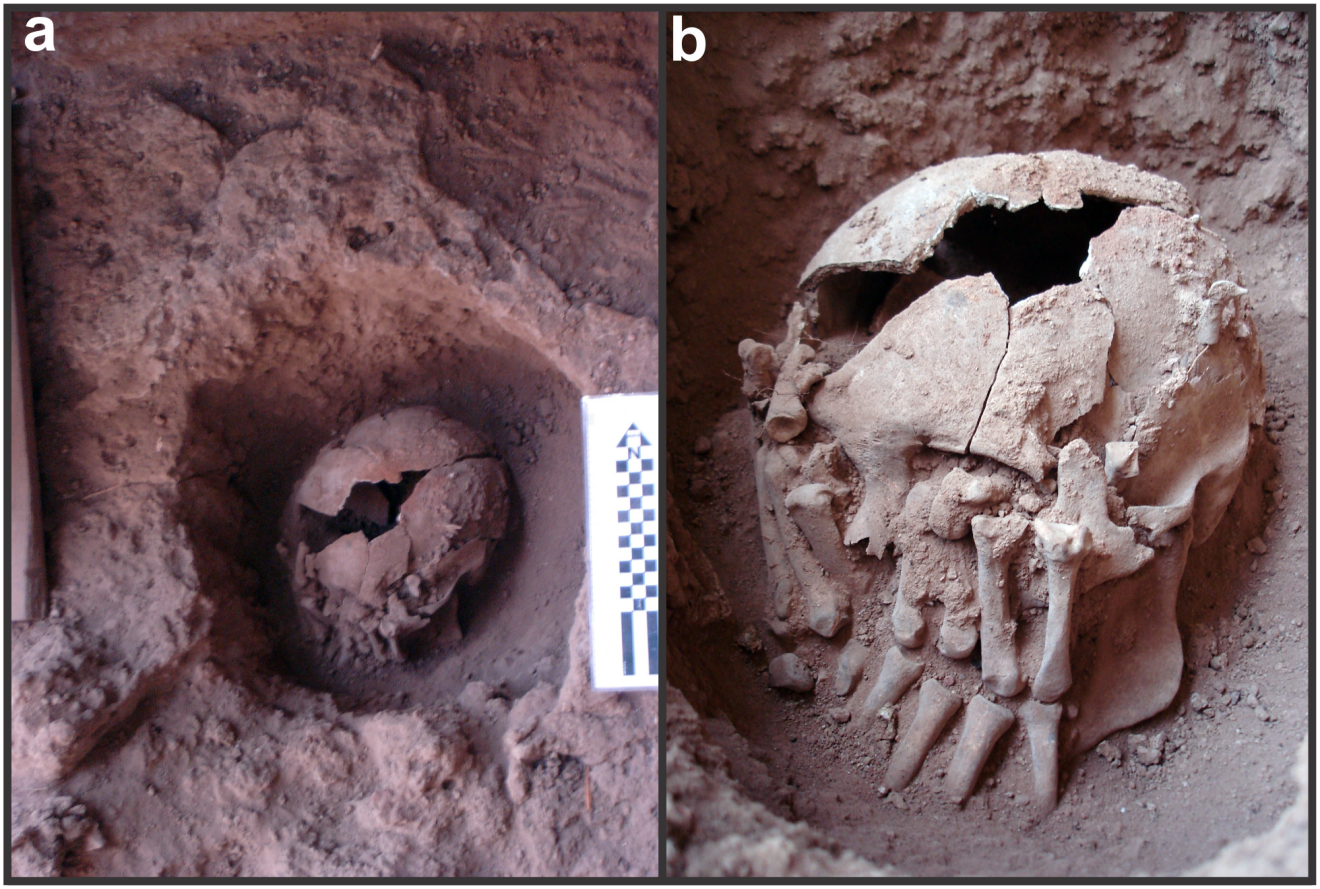
Burial 26. a) Pit shape; b) Arrangement of the hands over the skull.

**Fig 9:**
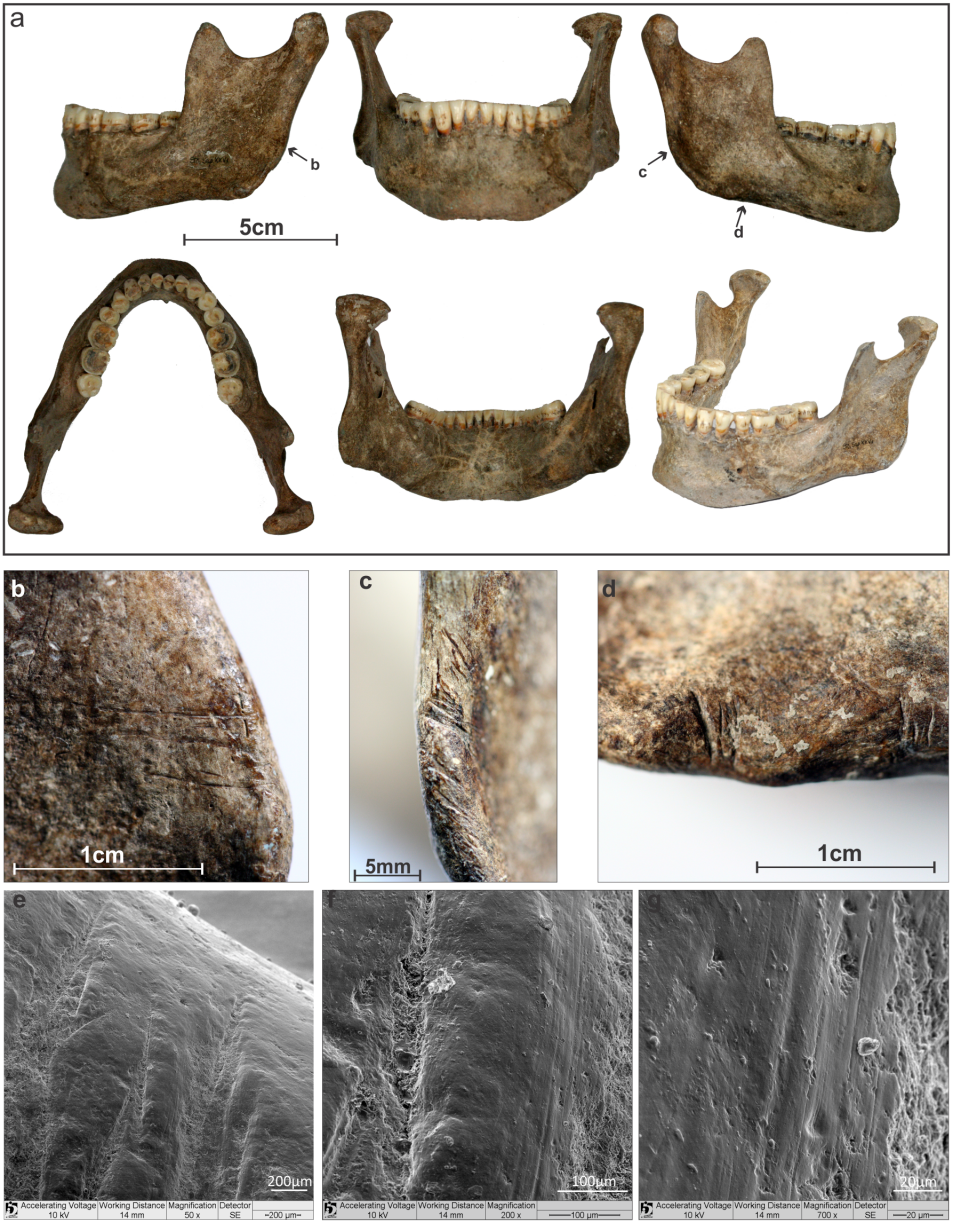
Mandible of Burial 26. a) The arrows point the location of the incisions; b) Incisions on the lateral surface of the left ramus; c) Incisions on the posterior margin of right ramus; e) Incisions in the lower margin of the right ramus; e); f) and g) SEM of the incisions on the inferior margin of the right ramus.

**Fig 10:**
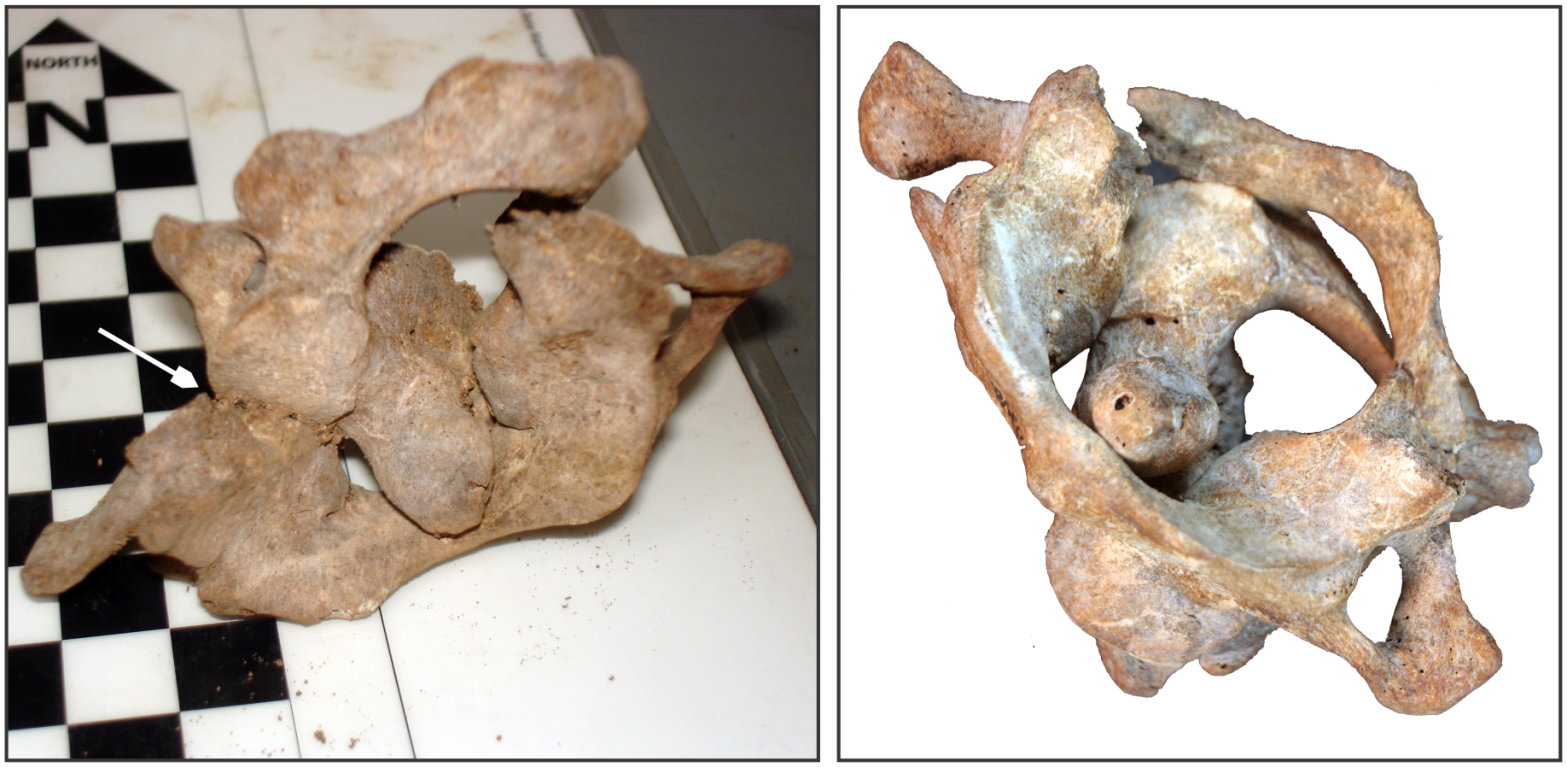
Atlas and axis of Burial 26. Although in anatomical position due the presence of carbonate cement, the posterior arch of the atlas was broken. a) Picture taken immediately after exhumation; the arrow indicates the point where the neural arch is attached to atlas by means of carbonatic concretion; b) Atlas was rotated 42 degrees in relation to the axis.

Several cut marks were observed on the cranial and vertebral elements of Burial 26 (see SI for a detailed description). The mandible showed a number of parallel cut marks on the inferior and posterior margins of the right ramus and on the posterior margin of the left ramus (Fig 9). Two parallel incisions were also identified on the right zygomatic bone. Concerning the neurocranium, a single vertical incision was found in the right side of the frontal bone. The incisions in the zygomatic and frontal bone are not, however, cut marks but result from taphonomic processes (see SI for cut mark analysis). In addition, parallel incisions were found near the mastoid angle of the right parietal bone and along the right lambdoidal suture of the occipital bone. The atlas and axis were cemented together by carbonate concretion in such an anatomical position that the C1 was rotated by 42° in relation to C2 (Fig 10). Two oblique and fibrous-like fractures were found in the atlas’ posterior arch, suggesting green bone breakage.

**Fig 11:**
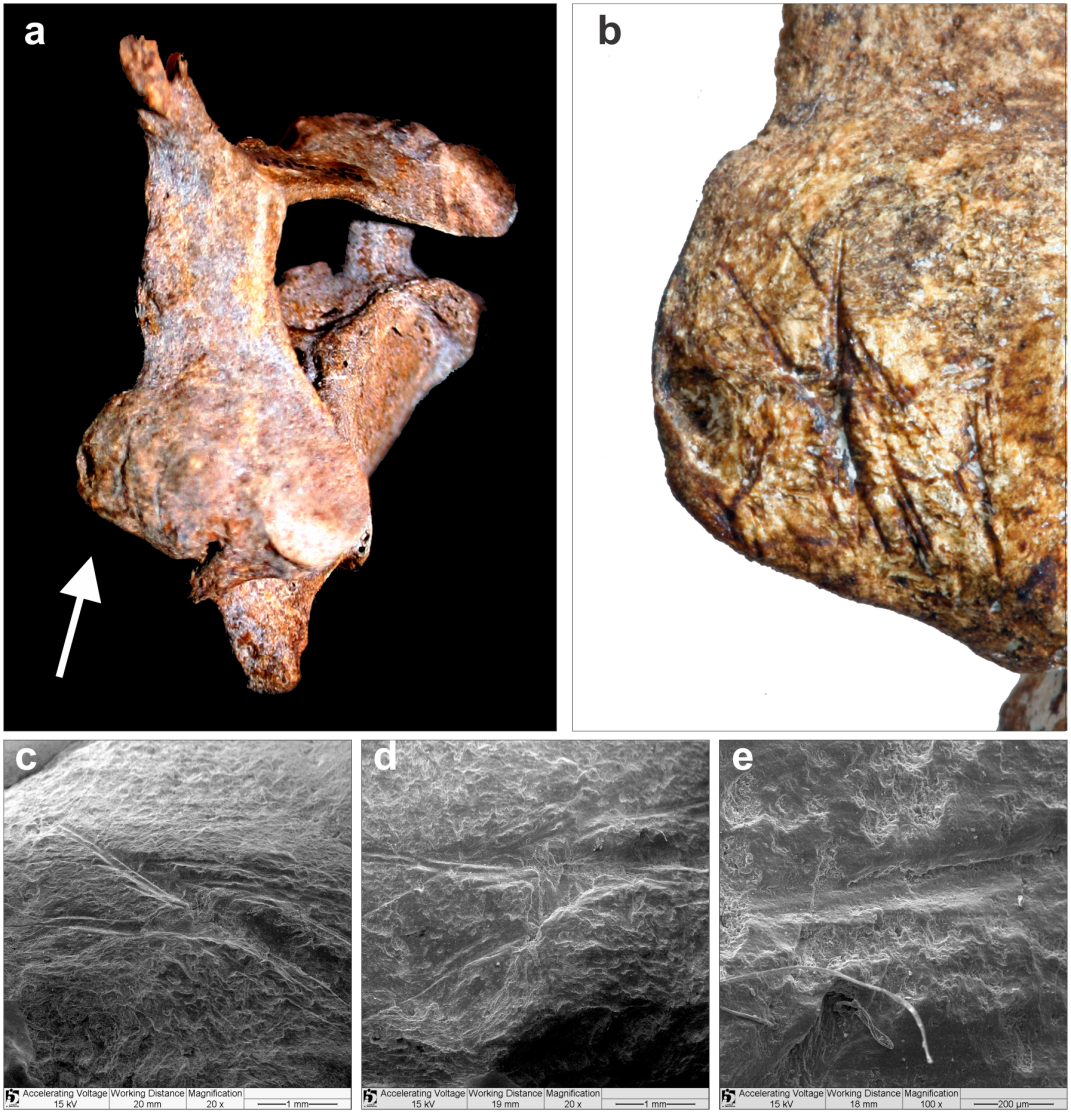
Burial 26’s sixth cervical vertebra. a) Carbonatic concretion was still present making the incisions in the column of the right articular processes, indicated by white arrow, very subtle; b) detail of the right column of articular processes after removal of concretion; c); d) and e) SEM of the incisions.

**Fig 12:**
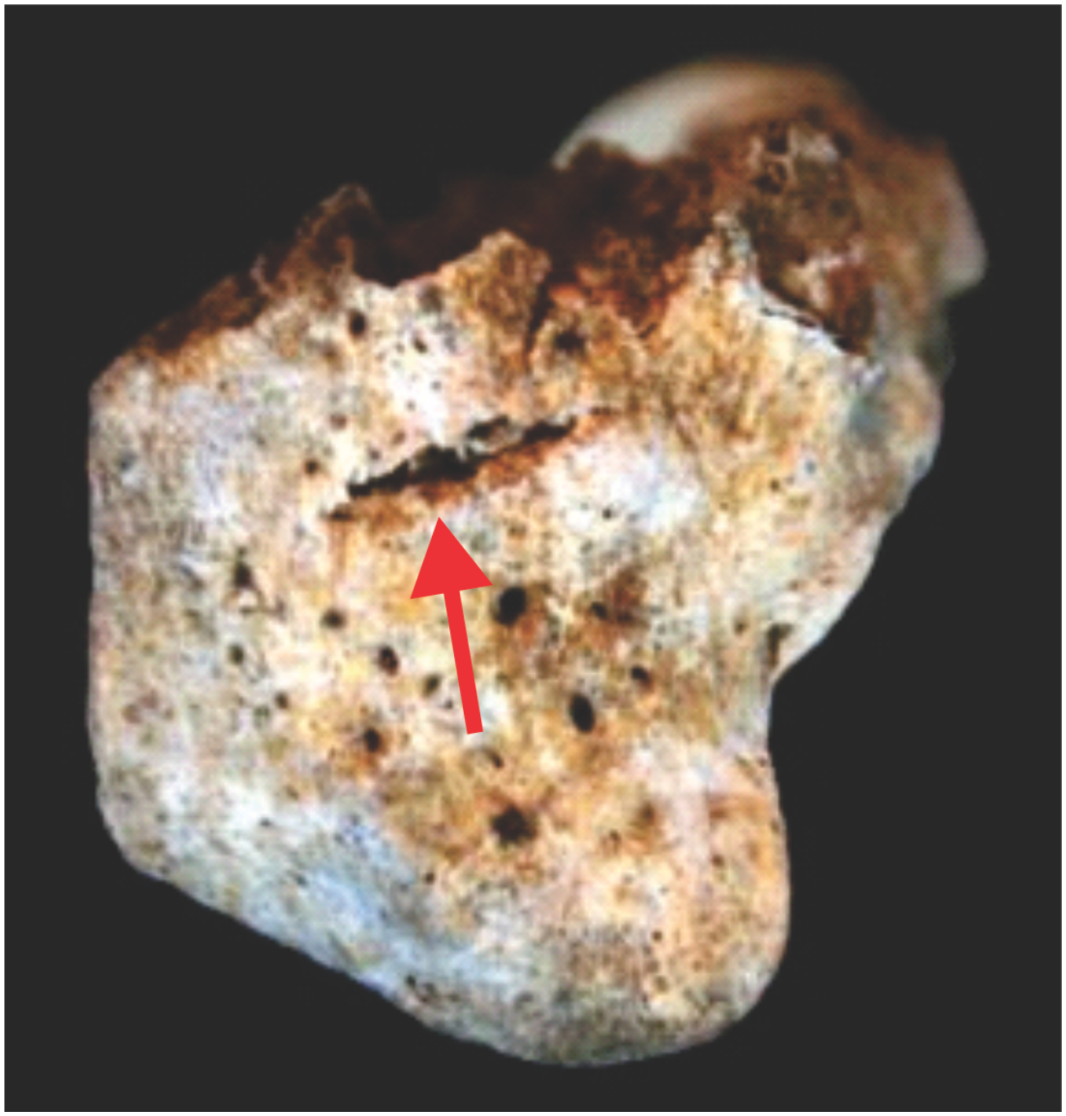
Distal extremity of the right radius. The red arrow points to the hack mark.

In the vertebrae, cut marks were observed at the right column of the articular processes of C6, where the zygopophysial joint capsule would be located (Fig 11). Concerning the hands, the distal segment of the right radius was clearly sectioned in a plane perpendicular to the long axis of the bone, as is made evident by a hack mark near the cut surface (Fig 12). These marks indicate that an implement was used to separate the hands forcibly from the arms. No cut marks were observed on the bones of the left hand, although the left radius and ulna were not recovered during the excavation.

Taken together, this assemblage suggests that two different procedures were applied to the skull of Burial 26: soft tissue removal and decapitation. Cut marks on the articular process of C6 point to the sectioning of the neck between C6 and C7. Cut marks on the posterior and inferior parts of the mandible are likely related to cutting of soft tissue in the floor of the mouth, the neck and the pharynx, respectively. The fracture of the atlas is in accordance with vertical pressure followed by hyperextension of the head [114], while the rotation of the atlas on axis may be related to head torsion. It is possible that multiple forces were applied to the head to detach it from the neck. Vault and zygomatic cut marks are attributed to soft tissue removal in the right side of the skull. Therefore, Burial 26 constitutes a clear case of decapitation (see SI).

### Dating

A fragment of the sphenoid from Burial 26 was pretreated at the Department of Human Evolution, Max Planck Institute for Evolutionary Anthropology (MPI-EVA), Leipzig, Germany, using the method described by Talamo and Richards [115]. The outer surface of the bone sample was first cleaned by a shot blaster and then 500mg of bone powder was removed. The sample was then decalcified in 0.5M aq. HCl at room temperature for about 4 hours, until no CO_2_ effervescence was observed. 0.1M aq. NaOH was added for 30 minutes to remove humic acids. The NaOH step was followed by a final 0.5M HCl step for 15 minutes. The resulting solid was gelatinized in a pH3 solution in a heater block at 75°C for 20h, following Longin et al., [116]. The gelatin was then filtered in an Ezee-Filter^TM^ (Elkay Laboratory Products (UK) Ltd.) to remove small (<8 μm) particles, and then ultra-filtered with Sartorius “Vivaspin 15” 30 KDa ultra-filters [117]. Prior to use, the filter was cleaned to remove carbon containing humectants [118]. The sample was then lyophilized for 48 hours.

C:N ratios, %C, %N, δ^13^C and δ^15^N values were measured at the MPI-EVA using a Thermo Finnigan Flash EA coupled to a Delta V isotope ratio mass spectrometer. For acceptable quality collagen, the atomic C:N ratio should be between 2.9 and 3.4 and a collagen yield of more than 1% of weight [119–121]. For Burial 26, the isotopic results, C:N ratios and collagen values are well within the accepted ranges (Table 1). The samples provided enough collagen for radiocarbon dating and were sent to the Klaus-Tschira-AMS facility of the Curt-Engelhorn Centre in Mannheim (MAMS), Germany, where they were graphitized and dated [122]. The resulting date was corrected for a residual preparation background estimated from pretreated ^14^C-free bone samples, kindly provided by the Oxford Radiocarbon Accelerator Unit (ORAU). The radiocarbon dates were calibrated using OxCal 4.1 [123] and SHcal13 [124] (Table 1).

**Table 1:** Isotopic values, C:N ratios, amount of collagen extracted (%Coll) refer to the >30 kDa fraction. δ^13^C values are reported relative to the vPDB standard and δ^15^N values are reported relative to the AIR.

MPI Code	Type	%coll	δ^13^C	δ^15^N	%C	%N	C:N	AMS Nr	^14^C Age	1σ err	Cal BP 68.2%	Cal BP 95.4%
S-EVA 26436	Sphenoid fragment	0.81	-19.03	5.86	3.00	1.17	3.00	MAMS-16368	8331	44	9146–9407	9127–9438

In addition to the date obtained at the MPI-EVA, another date was obtained from Beta Analytic. Despite the excellent preservation of Burial 26, small fragments of bone from the nasal cavity and sphenoid could not be reassembled to the cranium. A portion of 8.707 grams of this highly fragmented material was sent to Beta Analytic Laboratories in Miami in December 2008 (Beta# 253511). The final age result was 8540±50 ^14^C BP, the calibration age range was obtained with OxCal 4.1 [123] and SH13 [124] which resulted in an interval between 9.47 and 9.54 cal kyBP (68.2%) and between 9.43 and 9.55 cal kyBP (95.4%). Since the date from the Beta Analytic did not follow the same quality control parameters we adopted for bones at the MPI-EVA, we consider the latter as more accurate for dating Burial 26.

### Strontium isotopic analysis

Strontium isotopic analysis (^87^Sr/^86^Sr) of skeletal material is commonly employed to detect geographic provenance and mobility among mammals, including humans [125,126], because tooth enamel from individuals records the isotopic signal of when it was formed during the earliest stages of life, whereas bone isotopic signal reflects a period closer to the time of the death of the individual [127]. Since radiogenic isotope ^87^Sr forms by radioactive decay from rubidium (^87^Rb), the ^87^Sr/^86^Sr signature of a specific location is determined by the underlying bedrock age and its content of Rb. Younger geological formations like volcanic rocks have lower ^87^Sr/^86^Sr values than older geological formations such as granite. A specific geological strontium signature is incorporated into hard body tissues by direct substituting for calcium [125,128,129], since strontium enters the ecosystems without fractionation [130,131].

Among skeletal tissues, tooth enamel is the preferred substrate for this analysis, due to its greater resistance to diagenesis in the burial environment [132,133]. Within a single archaeological population, ^87^Sr/^86^Sr analyses of individuals’ teeth can potentially detect those who were born in the same geological substrates (“locals”) and those who were born in different geological substrates (“non-locals”). However, environmental background studies are needed to assess the local bioavailable ^87^Sr/^86^Sr signature from the different geologies in the study region [125,134] in order to assess possible provenance and mobility. The use of strontium isotopes to investigate questions relating to the identity (local versus foreign) of disembodied heads is a well-established field in the Andes [32,33,68,135].

**Fig 13:**
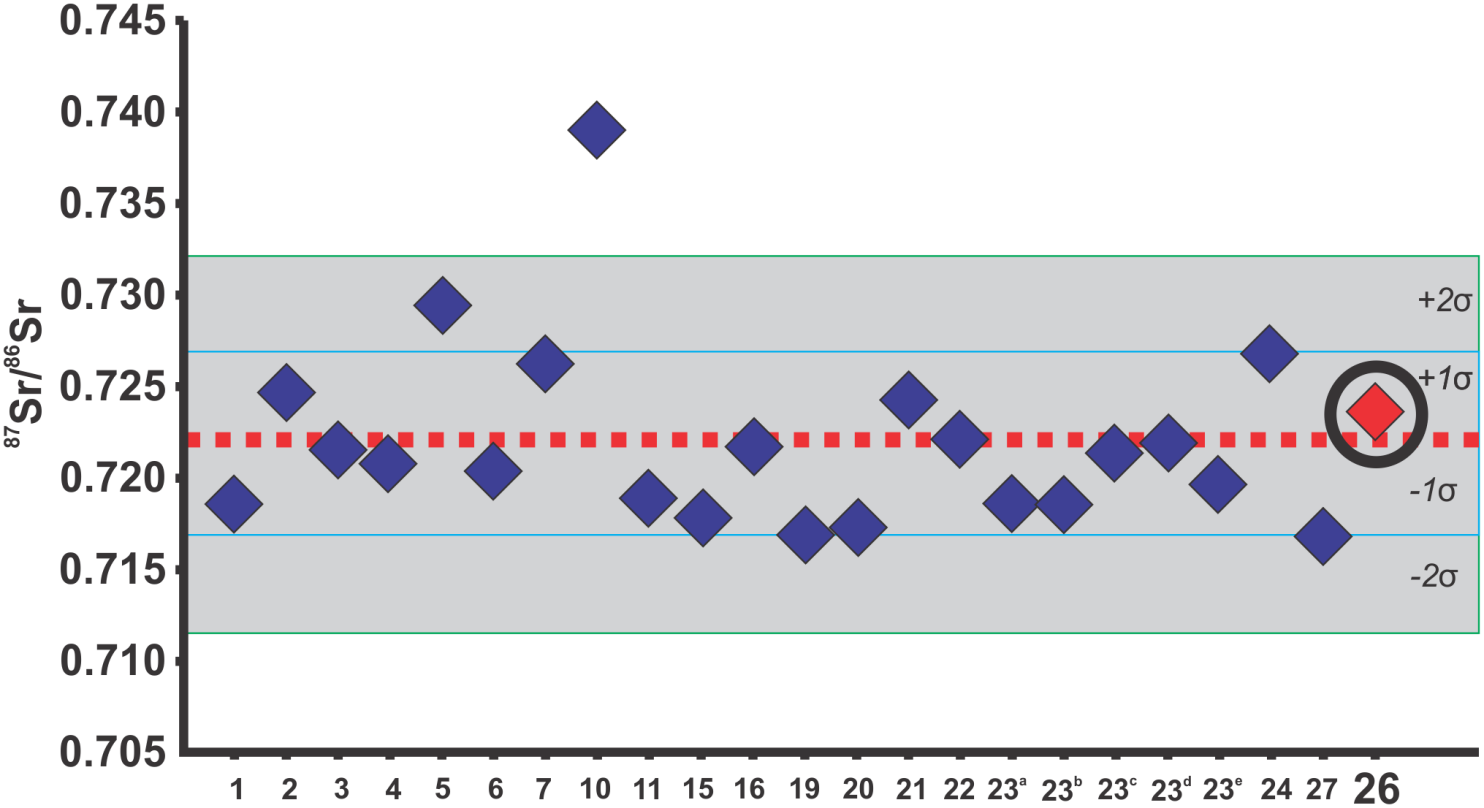
Strontium isotopic analysis. ^87^Sr/^86^Sr ratio enamel values from the individuals of Lapa do Santo, plotted on ^87^Sr/^86^Sr mean ratio value (red dashed line), mean ratio ± 1σ values (area between blue lines), and mean ratio ± 2σ values (area between green lines) of the entire sample. A black circle marks the decapitated individual.

Strontium ^87^Sr/^86^Sr values from 23 enamel samples were successfully measured (S1 Table; see SI for methodological details). The ^87^Sr/^86^Sr ratio measured in human enamel has a mean value of 0.722 ± 0.005 (1σ) and ± 0.001 (2σ), with minimum and maximum values of 0.717 and 0.739 respectively (Table 2). The value from the decapitated human Burial 26, (0.724) falls well within the 1σ range of the population (Fig 13), suggesting that at the time of its lower right P2 crown formation (3.6–6.6 years old [136]) this individual lived in a locality with similar strontium isotope values as the region where most of the others individuals of the population lived during their childhood, and therefore he was probably a local individual.

**Table 2:** S-EVA number, archaeological code, ^87^Sr/^86^Sr ratio, ^84^Sr/^86^Sr ratio, Sr concentration (ppm) and voltage (^88^Sr) from enamel of the human teeth prepared in solution and analyzed in the MC-ICP-MS.

S-EVA	Bur. #	Tooth	Start mass (mg)	^87^Sr/^86^Sr	^84^Sr/^86^Sr	Sr conc (ppm)	88Sr (V)
26019	1	Inferior Right M3	23.2	0.719	0.0565	123.5	15.7
26020	2	Superior Right P4	10.4	0.725	0.0565	181.5	15.7
26021	3	Inferior Right P4	33.9	0.722	0.0565	41.4	17.5
26022	4	Inferior Right dM2	21.7	0.721	0.0565	58.1	15.7
26023	5	Superior Right M3	24	0.729	0.0565	169.9	18.4
26024	6	Inferior Left dM2	23	0.720	0.0565	69.3	15.9
26025	7	Inferior Left dM2	20.9	0.726	0.0565	87.3	18.1
26026	10	Inferior Right P4	29.3	0.739	0.0564	123.3	18.0
26027	11	Inferior Right P4	15.1	0.719	0.0565	152.9	16.4
26028	15	Inferior Right P4	21.4	0.718	0.0564	155.4	18.2
26029	16	Inferior Right P4	24.8	0.722	0.0565	82.8	17.1
26030	19	Inferior Left dM2	19.7	0.717	0.0564	88.7	17.4
26031	20	Inferior Left dM2	16	0.717	0.0565	136.7	18.2
26032	21	Inferior Left M2	21.3	0.724	0.0564	99.7	21.3
26033	22	Inferior Right P4	34.5	0.722	0.0564	122.6	21.2
26034	23a	Inferior Right dM2	19.9	0.719	0.0565	65.1	21.6
26035	23b	Inferior Right dM2	9.2	0.719	0.0565	126.5	19.4
26036	23c	Superior Right P4	16.7	0.721	0.0564	216.5	20.1
26037	23d	Superior Right P4	14.3	0.722	0.0565	171.4	20.5
26038	23e	Inferior Left P4	13.4	0.720	0.0565	96.3	21.5
26039	24	Superior Right P4	9.5	0.727	0.0565	105.8	16.8
26041	27	Inferior Left dM2	20.6	0.717	0.0565	113.5	19.6
26040	26	Inferior Right P4	18.9	0.724	0.0564	163.8	19.4

### Morphological affinities

A complementary approach to strontium isotope in determining whether Burial 26 was a local or foreigner involves determining its genetic resemblance with the other individuals from Lapa do Santo. If genetically more distinct from the other individuals than the average, this would be compatible with Burial 26 being a foreigner to that group. Molecular data, however, is not yet available for the individuals from Lapa do Santo. Alternatively, cranial morphology can be used as a proxy to infer genetic relationships (see [51] for an analogous application of the method using dental traits), since there is a close link between cranial morphology and population history. This association was first recognized by studies demonstrating that craniometric traits, as many other phenotypic traits, present a moderate heritability [137–145], even though the heritability of each craniometric trait can vary considerably [143,145]. Under this assumption, genetic information can be estimated from phenotypic traits determined, at least partially, by quantitative genetic loci [144,146–152].

Linear measurements were extracted from the 3D digital cast of Burial 26 using Landmark 3.0. Linear measurements followed Howells protocol to allow the comparison of this specimen with Howells series [153,154], as well as Lagoa Santa and Colombian remains [78,155]. Only landmarks that could be easily identified in the cast were used for measurements. Measurements that required projections (e.g., maximum cranium breadth) were not taken, due to the difficulties to achieve similar results from measurements with calipers. In total, 24 of Howells variables were extracted from the virtual cast (S2 Table). However, the skull had an unusually long frontal (FRC) and high skull (BBH), outside of the 99% confidence interval of modern humans. Therefore, these variables were removed and all analyses were performed with the remaining 22 variables. Although the Howells database includes series from all continents, we selected here only the series from the Americas, Asia and Australo-Melanesia, due to its demonstrated relationship with the Lagoa Santa remains (e.g., Hubbe et al., 2010 [156]). Including series from regions that had no direct biological relationship with the Americas would add noise to the analyses, rendering the morphological affinities between Burial 26 and the other series harder to assess.

Since Burial 26 is a male, comparisons were made only with male specimens of the reference database. Only specimens that had at least 75% of the variables present were included in the analysis. This reduced the sample size of early Lagoa Santa and Archaic Colombia remains, but it minimized the frequency of missing values in the data (less than 6% of the total measurements in each series; S3 Table). Missing values were replaced via multiple regressions, following the same protocol and reasoning adopted by Hubbe et al. [156].

Analyses were performed on the raw measurements and subsequently on the measurements corrected for size differences between specimens. Size correction was accomplished by dividing each measurement by the geometric mean of the individual [157]. The geometric mean was also used as a proxy to overall cranium size of the individuals. All analyses were done for the original and the size corrected data. Burial 26 was compared to the reference series via a series of multivariate analyses.

Initially, to check if Burial 26 showed an unusual size, its geometric mean was compared to the geometric means of other Lagoa Santa remains, via a box-plot. Secondly, we compared its morphological affinities using a principal component analysis (PCA), based on the overall correlation matrix between the variables. PCA was calculated using the individual data and Burial 26 morphological affinities was contrasted with the 95% confidence ellipsis of the comparative regions according to the first two PCs. To simplify the reading of the plots, series were grouped according to their geographic regions (S3 Table).

Finally, Burial 26 was included in a Discriminant Functions Analysis (DFA) and classified according to its posterior probabilities to the comparative series. To complement the posterior probabilities, typicalities based on the Mahalanobis distances between Burial 26 and each of the reference series centroids were also calculated. All statistical analyses were performed in Statistica 7 (Statsoft Inc).

**Fig 14:**
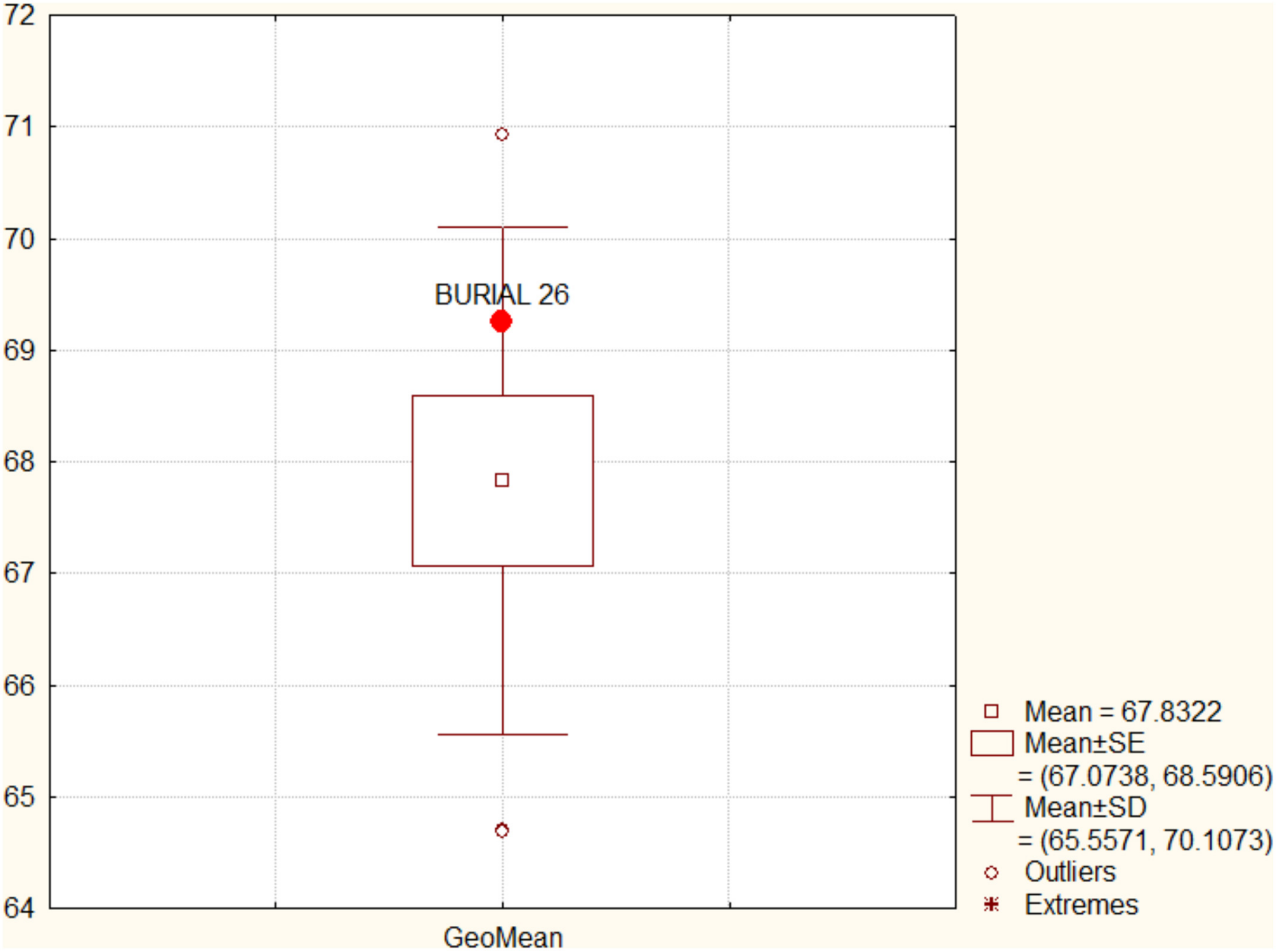
Boxplot of the geometric mean of Burial 26 compared to Lagoa Santa skulls.

**Fig 15:**
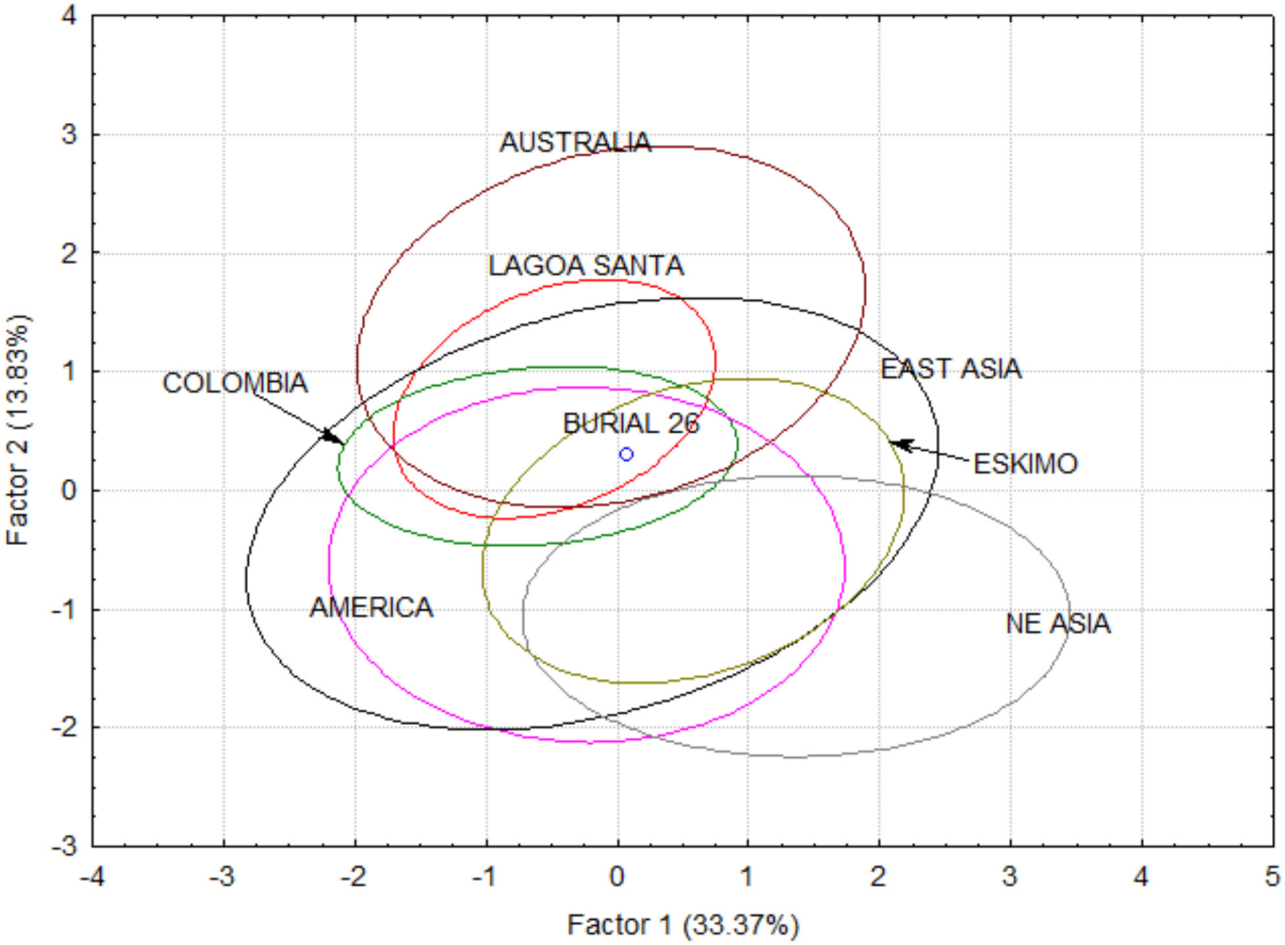
Morphological affinities of Burial 26 compared to the variation of the reference series, based on original variables (size and shape).

**Fig 16:**
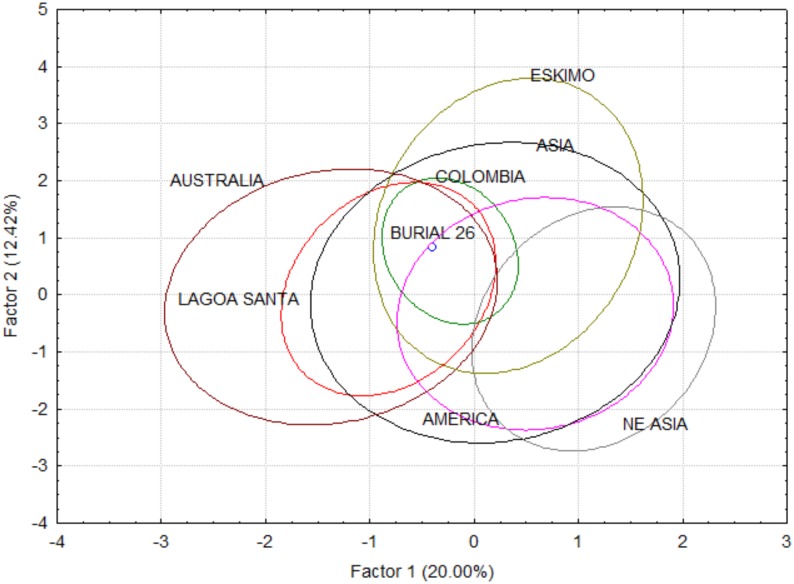
Morphological affinities of Burial 26 compared to the variation of the reference series, based on size corrected variables (shape alone).

The boxplot comparing the overall size of Burial 26 to other Lagoa Santa crania can be seen in Fig 14. Although above average in size, Burial 26 falls well within the distribution of Lagoa Santa. The PCA analysis of the raw data (Fig 15) and size corrected data (Fig 16) show similar results. In both plots, Burial 26 occupies a central position in the morphospace, falling inside the confidence ellipses of Lagoa Santa, Archaic Colombia and many of the comparative series included here.

The DFA also show similar results for both size and shape, and size corrected analyses (S4 Table). When either posterior probabilities or typicalities are taken into account, Burial 26 classifies clearly with Australia, which has been shown in the past to share high morphological affinities with Early South Americans [155,156]. Yet, interestingly, in none of the analyses Burial 26 appear close to the other Lagoa Santa remains. When typicalities are taken into account, in both analyses, Burial 26 is statistically different (p<0.05) from Lagoa Santa’s centroid. However, these results may be influenced in this case by the reduced number of individuals in the Lagoa Santa sample, which is probably biasing the population estimates in these analyses.

The results do not indicate Burial 26 from Lapa do Santo presents a distinct morphology compared to other specimens from the Lagoa Santa region, thus supporting the notion he was a local individual and not an outsider.

## Discussion

The early Holocene age of Burial 26 extends the timeline of decapitation in South America by more than 4500 years. As far as we could evaluate, in North America the oldest reported cases include the inferred decapitation from Windover Pond, Florida (8120–6990 cal BP)[158] and the demonstrated cases from the tributaries of the Ohio River in Illinois, Kentucky and Tennessee (6000–3000 cal BP)[159–161], which are also younger than Burial 26 from Lapa do Santo.

Geographically, the archaeological record of North America and Mesoamerica shows a more widespread occurrence of decapitation compared to South America, with cases occurring from the Arctic to southern Mexico[1]. Our findings suggest that South America had the same spatially widespread distribution observed for North America, making the occurrence of decapitation widespread across the whole continent since the beginning of the Holocene. In addition, they confirm that the vast territorial range of decapitation behavior described in ethnohistorical and ethnographic accounts for the New World has deeper chronological roots. Until now, every archaeological site in South America where evidence of decapitation was observed was related to the so-called Pan-Andean societies. Lapa do Santo, located in the lowlands of east-central South America, indicates that decapitation does not necessarily have a restricted Pan-Andean distribution.

Although the Eurocentric view has always understood decapitation in the context of inter-group violence, the archaeological and ethnographic record points to a more complex scenario in the New World [82]. In some cases, decapitation and the subsequent public exhibition of the severed head was indeed used as a punitive mean to subjugate rebellious groups (e.g., European colonizers and Inca). In some occasions, decapitation was just one among several other means of mutilating defeated enemies as part of sacrificial rituals and the disembodied head received little or no attention (e.g., Plaza 3A and 3C of Huaca de la Luna, Pacatnamu). In other cases, the heads of the enemies themselves were the main reason behind decapitation and they would be further transformed into valuable objects. Beyond memorializing victory those trophy heads were also symbolically embedded with signs of fertility and rebirth (e.g., Jivaro, Munduruku, Nazca). The commoditization of human heads was also common as part of an ancestral cult where the beheaded one was not the enemy but instead a member of the group (e.g., Asia 1, Aguazuque). The focus around the head or the skull would sometimes result in the explicit transformations of those body parts into material culture (e.g., the jar’s skulls from the Incas or Moches). Decapitation was not the only mean of obtaining a human head or skull. In some cases, usually related to ancestral cults, they were removed from previously interred individuals in advanced stages of decomposition.

Although no straightforward method is available to determine the nature of a severed head, the analysis of its context can provide relevant information. Trophy heads, for example, usually present the drilling of the skulls for carrying, or enlargement of the foramen magnum for brain removal [162]. At Lapa do Santo, neither drill holes nor an enlargement of the foramen magnum were observed in the skull, making it unlikely that this was a trophy head.

Determining the identity of the decapitated individual can also contribute to understanding the broader cultural context in which decapitation practices are inserted. A common parameter used in this task is the demographic profile of the samples. It is usually assumed that a sample composed of young males is more likely to reflect the execution of a group of defeated warriors instead of regular mortuary practices. Burial 26 was a young male. However, in the absence of other decapitated individuals in Lapa do Santo, it is hard to determine whether this indeed reflects a regional pattern.

The status of Burial 26 as a local or an outsider to the group is another relevant point. If an outsider, he might in fact represent an enemy. If local, he could represent an individual of unique status in the groups, like a venerated ancestral [30,66,135]. The results of the strontium isotope analysis for Lapa do Santo show a very similar ^87^Sr/^86^Sr value to almost all other individuals, offering no support to the notion that Burial 26 was an outsider. Additionally, the cranial morphological affinities of Burial 26 compared with other specimens from the same region provide no evidence that he was an outsider. Together with the osteological evidence indicating low levels of inter-group conflict in Lagoa Santa during the early Holocene [103], the result from the strontium isotope analysis is compatible with a scenario in which the ritualized decapitation of Burial 26 was not a violent act against the enemy but instead part of a broader set of mortuary rituals involving a strong component of manipulation of the body. The careful arrangement of the hands over the face is compatible with an important public display component in the ritual that could have worked to enhance social cohesion within the community. This ritualized burial attests to the early sophistication of mortuary rituals among hunter-gatherers in the Americas. In the apparent absence of wealth goods or elaborate architecture, Lagoa Santa’s inhabitants seemed to be using the human body to reify and express their cosmological principles concerning death. A more detailed evaluation of this matter will depend on further work in the region. After all, the findings at Lapa do Santo opens the possibility that similar practices occurred in other parts of east South America among other early Holocene hunter-gatherer societies.

## Supporting Information

S1 Fig Cranium of Burial 26. (TIF)

S2 Fig Frontal bone of Burial 26. a) Picture of the right region of the frontal bone. The arrows indicate the incision; b); c) and d) SEM of the incision.
(TIF)

S3 Fig Confocal image of the incision located in the frontal bone (same as depicted in Fig 6). a) Three-dimensional model (above) and topography (bottom) based on 20x lens. The white dotted rectangle delimits the area shown in “b”; b) Three-dimensional model (above) and topography (bottom) based on 50x lens. Note how the incision has a flat bottom not compatible with a cut mark.
(TIF)

S4 Fig Right malar of Burial 26. Yellow arrows indicate the very thin incisions on the zygomatic bone.
(TIF)

S5 Fig SEM and confocal microscopy of the incisions (green and white arrows) observed in the right zygomatic. (TIF)

S6 Fig Right asterionic region of the cranium of Burial 26. a) Picture of the posterior right portion of the cranium where incisions are present near the right asterion. b) Detail of the same area.
(TIF)

S7 Fig SEM of the right asterionic region of the cranium of Burial 26 (same as in S6 Fig). In low magnification (“a” and “b”), it is possible to observe the sub-parallel orientation of the possible cut marks (indicated by the green arrows). In higher magnification some incisions look more like v-shaped incisions (“c” and “d”) while others look more like broad striation (“e” and “f”).
(TIF)

S8 Fig Cervical vertebrae. They were complete and presented no signs of fracture or breakage.
(TIF)

S1 Table Operation parameters for MC-ICP-MS solution analysis used at the Max-Planck Institute for Evolutionary Anthropology (Leipzig, Germany). (DOCX)

S2 Table Craniometric variables used in this study. (DOCX)

S3 Table Comparative series included in the craniometric analyses. (DOCX)

S4 Table Classifications of Burial 26 according to Discriminant Function Analysis. (DOCX)

S1 Text Supplementary text containing a detailed description of Burial 26 and technical aspects of the methods used in this study. (DOCX)
