# Supplementary material for: The Oldest Case of Decapitation in the New World (Lapa do Santo, East-Central Brazil)
Source: PLoS One. 2015 Sep 23;10(9):e0137456. doi: 10.1371/journal.pone.0137456 (PMC4580647; doi:10.1371/journal.pone.0137456)
Supplement: S1 Table — (DOCX) [file pone.0137456.s009.docx]

**Table S1.** Operation parameters for MC-ICP-MS solution analysis used at the Max-Planck Institute for Evolutionary Anthropology (Leipzig, Germany).

| MC-ICP-MS | Thermo Fisher Neptune^TM^ |
| --- | --- |
| Forward power | 1200 W |
| Reflected power | <4 W |
| Interface cones | Nickel |
| Sample cones | Nickel |
| Skimmer cones | Nickel (X-cone) |
| Coolant argon gas flow | 15 L/min |
| Auxiliary argon gas flow | 0.8 L/min |
| Sample gas Argon gas flow | 1.17 L/min |
| Mass resolution | Low (400) |
| Lens settings | Optimized for maximum signal intensity |
| Nebulizer | Elemental Scientific Inc., Microflow 100μL/min, perfluoroalkoxy (PFA) |
| Sensitivity on ^88^Sr | 50 V/ppm |
| Cup configuration | L4 (^82^Kr); L3 (^83^Kr); L2 (^84^Sr); L1 (^85^Rb);  Ax (^86^Sr); H1 (^87^Sr); H2 (^88^Sr) |
| Data collection | 1 block, 50 cycles, 2 s integrations |
