# Supplementary material for: The Oldest Case of Decapitation in the New World (Lapa do Santo, East-Central Brazil)
Source: PLoS One. 2015 Sep 23;10(9):e0137456. doi: 10.1371/journal.pone.0137456 (PMC4580647; doi:10.1371/journal.pone.0137456)
Supplement: S2 Table — (DOCX) [file pone.0137456.s010.docx]

**Table S2.** Craniometric variables used in this study.

| **Variables included^a^** |
| --- |
| Basion-Nasion length (BNL) |
| Basion-bregma height (BBH)^b^ |
| B-zygomatic breadth (ZYB) |
| Biauricular breadth (AUB) |
| Minimum cranial breadth (WCB) |
| Biasterionic breadth (ASB) |
| Basion-prosthion length (BPL) |
| Nasion-prosthion height (NPH) |
| Nasal height (NLH) |
| Orbit height (OBH) |
| Orbit breadth (OBB) |
| Bijugal breadth (JUB) |
| Nasal breadth (NLB) |
| Palate breadth, external (MAB) |
| Bizygomaxillare breadth (ZMB) |
| Bifrontomallare breadth (FMB) |
| Biorbital breadth (EKB) |
| Interorbital breadth (DKB) |
| Malar length, inferior (IML) |
| Malar length, superior (XML) |
| Foramen magnun length (FOL) |
| Frontal cord (FRC)^b^ |
| Parietal cord (PAC) |
| Occipital cord (OCC) |

^a^ - measurement definitions according to Howells (1973, 1989)

^b^ – measurements not included in the analyses, due to outlier values in Burial 26.
