# Supplementary material for: The Oldest Case of Decapitation in the New World (Lapa do Santo, East-Central Brazil)
Source: PLoS One. 2015 Sep 23;10(9):e0137456. doi: 10.1371/journal.pone.0137456 (PMC4580647; doi:10.1371/journal.pone.0137456)
Supplement: S3 Table — (DOCX) [file pone.0137456.s011.docx]

Table S3. Comparative series included in the craniometric analyses.

| Series | Region | Sample Size | % of missing values |
| --- | --- | --- | --- |
| Lagoa Santa | Early South America | 9 | 4.04 |
| Sabana de Bogotá | Early South America | 14 | 7.14 |
| Peru | South America | 55 | - |
| Arikara | North America | 43 | - |
| Santa Cruz | North America | 51 | - |
| Eskimo | North America | 53 | - |
| Ainu | East Asia | 48 | - |
| Anyang | East Asia | 42 | - |
| Atayal | East Asia | 29 | - |
| Hainan | East Asia | 45 | - |
| North Japan | East Asia | 55 | - |
| South Japan | East Asia | 50 | - |
| Buriat | Northeast Asia | 55 | - |
| Australia | Australo-Melanesia | 52 | - |
| Tasmania | Australo-Melanesia | 45 | - |
| Tolai | Australo-Melanesia | 56 | - |
