# Supplementary material for: The Oldest Case of Decapitation in the New World (Lapa do Santo, East-Central Brazil)
Source: PLoS One. 2015 Sep 23;10(9):e0137456. doi: 10.1371/journal.pone.0137456 (PMC4580647; doi:10.1371/journal.pone.0137456)
Supplement: S4 Table — (DOCX) [file pone.0137456.s012.docx]

Table S4. Classifications of Burial 26 according to Linear Discriminant Analysis.

|  | Size and Shape | | |  | Shape alone | | |
| --- | --- | --- | --- | --- | --- | --- | --- |
| Reference series | Posterior Probability | D^2^ | Typicality | Reference series | Posterior Probability | D^2^ | Typicality |
| Australia | 0.902 | 22.779 | 0.356 | Australia | 0.884 | 22.227 | 0.387 |
| Tolai | 0.031 | 29.694 | 0.098 | Santa Cruz | 0.036 | 28.606 | 0.124 |
| Eskimo | 0.024 | 30.054 | 0.091 | Tolai | 0.033 | 28.966 | 0.115 |
| Santa Cruz | 0.017 | 30.654 | 0.08 | Peru | 0.019 | 29.978 | 0.092 |
| Tasmania | 0.01 | 31.532 | 0.065 | Tasmania | 0.008 | 31.299 | 0.069 |
| Ainu | 0.007 | 32.207 | 0.056 | Ainu | 0.008 | 31.587 | 0.064 |
| South Japan | 0.003 | 34.458 | 0.032 | Atayal | 0.005 | 31.413 | 0.067 |
| Peru | 0.002 | 34.872 | 0.029 | South Japan | 0.004 | 33.164 | 0.044 |
| Lagoa Santa | 0.001 | 32.759 | 0.049 | Eskimo | 0.002 | 34.389 | 0.033 |
| Atayal | 0.001 | 35.215 | 0.027 | North Japan | 0.001 | 36.014 | 0.022 |
| North Japan | 0.001 | 37.021 | 0.017 | Arikara | 0.001 | 36.388 | 0.02 |
| Arikara | 0 | 37.554 | 0.015 | Hainan | 0 | 40.326 | 0.007 |
| Hainan | 0 | 43.366 | 0.003 | Lagoa Santa | 0 | 37.525 | 0.015 |
| Sabana de Bogotá | 0 | 41.882 | 0.004 | Anyang | 0 | 43.13 | 0.003 |
| Anyang | 0 | 44.947 | 0.002 | Sabana de Bogotá | 0 | 41.237 | 0.005 |
| Buriat | 0 | 52.443 | 0 | Buriat | 0 | 51.349 | 0 |
