# Supplementary material for: The Oldest Case of Decapitation in the New World (Lapa do Santo, East-Central Brazil)
Source: PLoS One. 2015 Sep 23;10(9):e0137456. doi: 10.1371/journal.pone.0137456 (PMC4580647; doi:10.1371/journal.pone.0137456)
Supplement: S1 Text — (DOCX) [file pone.0137456.s013.docx]

The oldest case of decapitation in the New World (Lapa do Santo, east-central Brazil)

(SUPPLEMENTARY INFORMATION)

André Strauss*^1^, Rodrigo Elias Oliveira^2^, Danilo V. Bernardo^2,3^, Domingo C. Salazar-García^1,4,5^, Sahra Talamo^1^, Klervia Jaouen^1^, Mark Hubbe^6,7^ , Sue Black^8^, Caroline Wilkinson^8^, Michael Phillip Richards^1,9^, Astolfo G. M. Araujo^2, 10^, Renato Kipnis^2, 11^, Walter Alves Neves^2^

^1^ Department of Human Evolution, Max Planck Institute for Evolutionary Anthropology, Leipzig, Germany

^2^ Laboratório de Estudos Evolutivos Humanos, Departamento de Genética e Biologia Evolutiva, Instituto de Biociências, Universidade de São Paulo, São Paulo, Brazil

^3^ Instituto de Ciências Humanas e da Informação, Universidade Federal do Rio Grande, Rio Grande, Brazil

^4^ Department of Archaeology, University of Cape Town, Rondebosch, South Africa

^5^ Departament de Prehistòria i Arqueologia, Universitat de València, València, Spain

^6^ The Ohio State University, Department of Anthropology, Columbus, USA

^7^ Instituto de Investigaciones Arqueológicas y Museo, Universidad Católica der Norte, San Pedro de Atacama, Chile

^8^ University of Dundee, Centre for Anatomy & Human Identification, Dundee, UK

^9^Department of Anthropology, University of British Columbia, Vancouver, Canada

^10^ Laboratório Interdisciplinar de Pesquisas em Evolução, Cultura e Meio Ambiente, Museu de Arqueologia e Etnologia, Universidade de São Paulo, São Paulo, Brazil

^11^ Scientia Consultoria Científica Ltda., São Paulo, Brazil

***** Corresponding Author: André Strauss

E-mail: andre_strauss@eva.mpg.de (AS)

**Supplementary Information**

**Archaeological context**

**Burial 26’s grave**

Burial 26 was located in the center of unit L11 (Fig. 7). At the same level other five burials were found in this unit (Burials 18, 20, 23 and 27). At the surface, the z-value at the center of the unit was 0.108. The z-value at the top of Burial 26 was -0.443, corresponding to the highest point of the biggest limestone block that was above the grave (see arrow in Fig. 7). The z-value at the top of the skull was -0.684 and at the base of the grave -0.684. Therefore, top and the base of Burial 26 were located, respectively, at 56 and 79 centimeters below the surface.

The grave was circular with a diameter of ca. 40 centimeters. It was excavated within the harder matrix of the site and filled by soft and friable sediment. Above the skeleton, five blocks of limestone of different sizes were deposited (Fig. 7). The blocks were completely within the grave boundaries.

**Sex and age at death estimation**

The sex of Burial 26 was estimated using the standard craniometric method described in Buikstra and Ubelaker [1]. Five traits were utilized to characterize its cranial morphology. Burial 26 scored 4 and 5 in all traits, indicating a male morphology. In addition, this cranium shows high general robusticity in relation to other skulls from the same site. Da-Gloria [2] estimated the age at death of this individual using permanent molar tooth wear. The method is an adaptation of the work of Miles [3]. Da-Gloria [2] used 30 sub-adult individuals (under 18 years old) from Lagoa Santa as the population baseline. The ages of the sub-adults were estimated by Da-Gloria [2] using the dental developmental chart of Ubelaker [4] and tooth wear was scored using the method of Scott [5]. Instead of using the seriation process of Miles method, a best-fit regression curve was applied. The regression curve of the sub-adult baseline (molar wear versus age) was used to infer the unknown age of Burial 26. The method resulted in the age of 32 years old at death for Burial 26 [2]. The method of cranial suture closure was inapplicable to Burial 26 due to the lack of suture visibility.

**The Skull**

The cranium and mandible were in occlusion and facing southwest. The cranium was almost fully reassembled in the laboratory (Fig. S1). Incisions were observed in three different regions of the cranium. In the right side of the frontal bone a single incision of five centimeters long was observed (Fig. S2). This incision is very linear and homogeneous through its extensions, with parallel margins. It is also relatively wide and presents a transversal section that is “U” shaped. In the scanning electron microscope (SEM) (Fig. S2b-2d) and confocal microscope (CM) (Fig. S3), the flat bottom of this incision is evident and micro-striations are not present. In the right zygomatic bone two very thin and barely distinguishable incisions parallel to each other were observed (Fig. S4). Through SEM and CV microscopy (Fig. S5) it is possible to see that they present less than 80 microns breadth but do present a v-shaped profile. Finally, near the right asterion on the occipital and parietal bones, a profusion of sub-parallels incisions are present (Fig. S6). As can be in the SEM images (Fig. S7) these incisions are short with less than 1 centimeters of length and while some look like incisions with a v-shaped profile, others resemble broader striations.

The mandible was covered by a thin layer of calcium carbonate that was removed with the assistance of acetic acid solution in concentration of 10%. After removal, incisions were evident in the inferior and posterior margins of the right ramus and in the posterior region of the lateral surface of the left ramus (Fig. 10). Within each of these anatomical regions, the incisions occur in a sub-parallel cluster. They vary in width going from 0.05 to 0.1 millimeters. In the SEM images it is possible to observe very fine parallel striation within the incisions (Fig. 10), a diagnostic characteristic of stone flake cut marks.

**The Cervical Vertebrae**

Only the first six cervical vertebrae (C1-C6) were found. Except for the atlas (see below), the vertebrae show no signs of breakage or fracture (Fig. S8). They were articulated with each other (Fig. 8a-8b). However, the whole set of vertebrae was anteriorly dislocated. The atlas, for example, was not in direct articulation with the occipital condyles, but anteriorly dislocated by approximately two to three centimeters (Fig. 8c-8d). The third to sixth cervical vertebrae were located within the mouth and oriented perpendicular to the basicranium in such a way that C3 was very close to the palate and the body of the sixth cervical vertebra was located between the posterior part of the mandibular corpus (i.e., on the line between the molars, see Fig. 8a). The atlas and axis, on the other hand, were aligned perpendicularly to the coronal plane and, therefore, they were also perpendicular relative to the other cervical vertebrae (Fig. 8d). In addition, the atlas was rotated by 42º to the left with respect to the axis (Fig. 11b). They were found cemented to each other in this disposition (see Fig. 11a for field picture immediately after recovery) and were not separated later on during laboratory work. The posterior arch of the atlas was broken. Two oblique and fibrous-like fractures, typical of green bone breakage, characterize the breakage of the posterior arch (Figure 11).

Among the vertebrae, incisions were observed only at the right column of the articular processes of C6, where the zygopophysial joint capsule would be located (Fig. 12). These incisions were originally covered by carbonate cement and were very subtle (Fig. 12a). After treatment with acetic acid in concentration of 10%, the carbonate was removed and the incisions fully exposed (Fig. 12b). These incisions present a “V” shape transversal profile, are of no more than 1 cm of length and 0.5 cm of width. Parallel to the main grooves, very fine striation can be observed by naked eyes. These incisions are clearly cut marks made by flakes.

The hyoid bone was not found and there is no reason to postulate a taphonomic or post-depositional reason for this absence.

**The hands**

Both hands were found lying over the skull with the palmar surfaces in contact with the face. The right hand was laid over the left side of the face with distal phalanges pointing down (i.e., to the chin), while the left one was laid over the right side of the face with distal phalanges pointing up (i.e., to the forehead) (Fig. 9). All bones from both hands were found. The distal part of the right radius was the only bone present from the lower arm. In general, the bones were in direct anatomical articulation. Still, in the superior region of the grave, just above the calvaria, where the distal phalanges of the left hand and the wrist bones of the right hand were located, some perturbation was observed. A distal phalanx, for example, was cemented to the left parietal bone. The distal extremity of the radius was found within the left orbital cavity and some of the carpal bones of the right hand were within the left temporal fossa.

The distal extremity of the radius was clearly sectioned in a transversal plane a few centimeters before the distal end of the bone. A chop mark parallel to the sectioned surface can be observed in the lateral side of the bone (Fig. 13). On the hand bones, no incisions were observed.

**Decapitation process and soft tissue manipulation**

In the archaeological literature decapitations are classified as inferred or demonstrated. Demonstrated cases consist of injuries or mutilations which show unhealed cut marks, while inferred cases show headless or dismembered skeletons in undisturbed context but with no reported cut marks. Burial 26’s fully articulated nature and the presence of cut marks clearly indicate that this is a demonstrated case of decapitation. However, the scarcity of cut marks in the vertebrae does not correspond to the more flagrant cut marks usually associated with unequivocal cases of decapitation. The proper interpretation of this process depends on a great extent on defining how many cut marks are found and which of those cut marks are directly related to the process of decapitation.

The incisions in the mandible and in the sixth cervical vertebrae are clear evidence of cut marks made by stone flakes, as demonstrated by the morphology of the cut seen under the SEM and CM. Cut marks in the posterior region of the mandible are common on cases of decapitation. However, in such cases the plane of cut is usually much closer to the nuchal plane than in Burial 26, resulting in cut marks on the mastoid and mandible. In Burial 26, on the other hand, the cut plane was between the C6 and C7 at the shoulders height, well below the nuchal plane. As expected, there are no signs of cut marks near the mastoids. Still, one possible way of making the cut marks in the mandible compatible with the decapitation process is to postulate that the head was hyperflexed (chin touching the rib cage) when the cervical spine was cut. In this position, the ramus of the mandible would have been in the same plane as the last cervical vertebrae.

Even assuming that both cut marks in the sixth cervical vertebrae and in the mandible are directly related to the decapitation procedure, Burial 26 still shows few cut marks when compared to other unequivocal cases of decapitation reported in the literature. Two explanations for this scarcity of marks are 1) an advanced degree of decomposition of soft tissues minimized the necessity of cutting and/or 2) that the strategy adopted to remove the head was one that minimized the presence of cut marks. Concerning the first possibility, the absence of some bones like the hyoid and hand/wrist bones could be interpreted as supporting a somewhat advanced degree of decomposition. However, the distal and intermediate phalanges were articulated, showing that, at the moment of interment, even the most delicate labial articulations were still fully preserved. The articulations between the hand phalanxes are among the first parts of the human body to start decomposing [6]. A picture in which the soft tissues attached to the hyoid bone or the ones that connect the radius and ulna to the hand were decomposed while the soft tissues that hold the intermediate and distal phalanxes together is unlikely. Regarding the second explanation, it is possible that the procedure applied for removing the head was not solely based on cutting. This possibility finds support on the unique arrangement of atlas and axis (i.e., anterior dislocation in relation to the foramen magnum, fracture of posterior arch and rotation in relation to axis). One explanation is that the position of the atlas in relation to axis was the result of an excessive rotation of the head around the cervical axis and the fracture of the posterior arch as a consequence of vertical compression of the vertebral column followed by hyper-extension of the head [7]. Such extreme forces that are well beyond the normal anatomical limits are compatible with a scenario in which the head was pulled away. The relative importance of force and cutting to remove the head remains to be further investigated through experimental work on cadavers.

As a working hypothesis, we postulate that this case of decapitation involved two consecutive steps. First the cervical spine was exposed by the removal of the main muscles and ligaments of the neck and adjacent areas using cutting stone flakes. Muscles such as splenius capitis and sternocleidomastoid at the back of the head, and mylohyoid and digastric muscles between the hyoid and mandible were cut in the processes. This procedure resulted in the observed cut marks in the sixth cervical vertebrae and in the mandible. The separation of the head from the body, however, was not achieved by means of cutting instruments alone, but by pulling and rotational forces. These forces resulted in the last stage of individualization of the head, also causing the fracture of the atlas, its rotation in relation to axis and the anterior displacement of the vertebral column to the foramen magnum.

In addition to the process of decapitation, the incisions observed in the cranium might point to a secondary manipulation of the skull. If these incisions are indeed cut marks they are anatomically compatible with a process of soft tissue manipulation in the right side of the skull. Among the three regions of the cranium where incisions were observed the group near the right asterion is the one that most closely resembles cut marks associated with defleshing. They occur in the form of sub-parallels clusters and are “V” shaped in transversal section. The incisions in the frontal and in the zygomatic bone, on the other hand, do not present typical features of defleshing cut marks. The first one is broad, the margins are linear, there are no striations on the walls and the bottom is flat and smooth. These characteristics are not typical of marks made by flakes. Furthermore, there is a single incision, which is not compatible with the cluster of sub-parallels cut marks usually associated with defleshing cut marks.

The incisions in the maxilla are very thin, being incompatible with a process of substantial skin removal. Taken together, the morphology of the cut marks does not point to a single and uniform process of skin manipulation. Indeed, there is no undisputable evidence of defleshing of the skull. Experimental work needs to be done to determine the nature of the manipulation and the object utilized on the skull.

The chop mark in the right radius is a clear evidence that the amputation of the hands were achieved by sectioning the bone. However, the absence of both ulnas and the left radius, in one hand, and the absence of cut marks in any of the bones of the left hand point to a more complex scenario. Assuming these bones were not missing as a consequence of high levels of decomposition of the soft tissues, their absence might indicate that forceful movements (i.e., pulling, shearing and twisting) played an additional role in these dismemberments.

Finally, a comparison with a recent forensic case of decapitation supports that the case from Lapa do Santo is indeed a decapitation done while soft tissues were fully present and also points to the high levels of anatomical expertise demanded by the task. Before presenting the forensic case, however, it is important to keep in mind that none of the modern classifications for dismemberment or decapitation leaves room for the possibility of a ritual rationale that engenders respect, since according to modern law, mutilation of the deceased is a crime in most countries. In modern forensic investigations, dismemberment is classified into four general groups: defensive, aggressive, offensive and necromaniacal. Within these, defensive dismemberment is the most common representing an act undertaken to facilitate transportation of the remains, cover up traces of a crime or hinder identification of the deceased. Aggressive mutilation is where anger is expressed by the perpetrator on the victim after death. Offensive mutilation relates to a lust or necrosadistic murder and is performed usually to release sexual pressure or undertake sexual activities. Necromanical mutilation occurs when the perpetrator keeps a part of the remains as a trophy.

Therefore, to relate decapitation to modern practices is difficult with regards to motive but not necessarily with regards to the expertise of the perpetrator. Jeffrey Howe was murdered in the UK in March of 2009. His head was found in Leicestershire and his torso, his right leg, his left leg and his right forearm were each found in different locations throughout Hertfordshire. What was unusual about the dismemberment was the skill with which the body parts were removed, as if the perpetrator had training or extensive anatomical knowledge. In particular, the marks seen on the skull and the cervical vertebrae resonate with the remains found in Burial 26.

Removal of the head cleanly from the rest of the body is a difficult task as the overlapping nature of the vertebrae generally prevents a clean cutting action. Most dismemberments occur between C3 and C6 and in forensic cases are generally traumatic causing extensive bone splintering either through the use of a saw, an axe, a meat cleaver or some other heavy implement. However in the case of Jeffrey Howe (known colloquially as the Jigsaw Murder), the marks were restricted to areas on the mandible and the sides of the vertebral column and his hyoid was not found. Stephen Marshall was convicted of the murder and sentenced to life in prison. After sentencing, he admitted to being a cutter for a London drug gang, and he had dismembered many bodies and was therefore skilled in his trade. What he did was:

- Cut around the margins of the mandible and remove the floor of the mouth as one would in a *postmortem* examination.
- Reflect the tongue, pharynx and larynx away from the vertebral column via the pre-vertebral lamina of the cervical fascia (thereby removing the hyoid bone too).
- This allowed the position of the intervertebral discs to be seen anteriorly and permit a sharp blade to be inserted into the space between the two vertebral bodies.
- With a twisting of the neck, subluxation of the superior and inferior articular facets can occur, allowing the sharp blade to cut through the remaining soft tissue without resulting in extensive fracturing to either vertebra.

Therefore, in similarity with Burial 26, Jeffrey Howe showed cut marks around the inferior and posterior regions of the rami of the mandible. The hyoid bone was absent. Cut marks were noted only around the articular pillar region of the vertebral column in the region of separation between the two vertebrae where decapitation occurred. Twisting of the head to generate the subluxation of the joints can cause fracturing primarily of the C1 vertebra, which is attached firmly to the skull base. Further, there was removal of both the superficial and deep muscles of the face and back of the neck in the case of Howe’s murder, in an attempt to conceal other forensic evidence. Removal of the skin and muscles attached to the skull could result in the marks seen on the frontal, parietal, zygomatic and temporal bones seen in Burial 26. The features seen in the Jeffrey Howe and the Burial 26 cases are strikingly similar and suggest an element of skill and expertise in the decapitation process.

**Strontium isotopes**

The human teeth from Lapa do Santo were prepared and analysed for solution MC-ICP-MS strontium isotope analysis in the lab facilities of the Department of Human Evolution from the Max-Planck Institute for Evolutionary Anthropology (MPI-EVA) in Leipzig, Germany [8]. Solid pieces of enamel weighting approximately 20 mg were drilled from the crown of each of the teeth, spanning from the cement-enamel junction to the occlusal surface, and cleaned thoroughly on all sides under a magnifying lens with a diamond drill bit to ensure no dentine or other material remained attached to it. After the drilling and cleaning, the pieces of enamel were sonicated for at least 15 minutes in high purity deionized water, before they were taken to the MPI-EVA clean lab facility (PicoTrace GmbH, Bovenden, Germany). The samples were then rinsed three times with high purity deionized (18.2 MΩ) water (Milli-Q® Element A10 ultrapure water purification system, Millipore GmbH, Schwalbach, Germany), rinsed once with ultrapure acetone (GR for analysis grade, ≥ 99.8 %, Merck KGaA, Darmstadt, Germany), and dried overnight.

Further preparation of the enamel samples followed a modified version of the method described by Deniel and Pin [9]. Each enamel sample was weighed into clean 3 mL Savillex^TM^ (Minnetonka, MN, USA) vials and closed-vessel digested on a heating block at 120 ºC in 1 mL of 14.3M nitric acid (HNO_3_) before being evaporated to dryness at around 90-120 minutes. The resulting residue was then re-dissolved in 1 mL 3M HNO_3_ in order to pass its solution through ion exchange chromatography using 50-100 μm bead size Sr-spec^TM^ resin (EiChrom Technologies, Inc., Darien, USA) suspended in ultrapure deionized water [10] and previously cleaned following the procedure delineated by Charlier and collaborators [11]. Several washes were carried out with 3M HNO_3_ before the Sr in the sample was eluted in ultrapure deionized water, dried down, and re-dissolved in 3% HNO_3_ prior to MC-ICP-MS analysis.

A standard with known strontium isotope values (Bone Meal SRM 1486, National Institute of Standards & Technology, USA) and a blank sample were prepared parallel to the samples. Thus, one preparation batch was formed by 13 samples, 1 standard, and 1 blank. All acids used were made from SupraPur® grade (Merck KGaA) stock solutions and diluted using ultrapure deionized water.

A Thermo Fisher Neptune^TM^ (Thermo Fisher Scientific Inc., Dreieich, Germany) MC-ICP-MS instrument at the MPI-EVA facilities (see Table S1 for operational parameters) was used to obtain the strontium isotope measurements. This mass spectrometer is a high-resolution double-focusing one, equipped with nine Faraday detectors fitted with 10^11^ Ω resistors (four movable detectors H1-H4/L1-L4 on either side of a fixed axial detector) and a Virtual Amplifier^TM^ system which eliminates possible amplifier-detector bias and provides a dynamic range of 5 mV to 50 V on each detector [12,13]. A 100 μL/min self-aspirating capillary and MicroFlow PFA (perfluoroalkoxy) ST-nebulizer (Elemental Scientific Inc., Omaha, USA) was used to introduce the solutions, diluted in 3% HNO_3_ to give ^88^Sr signal intensities of 20-25 V into the plasma.

A static mode using a collector configuration similar to that described by Batey and collaborators [12] was used to measure ^87^Sr/^86^Sr strontium isotope values. The analysis of each sample was divided in two consecutive parts: a first baseline measurement at half mass positions (85.6 and 86.5) of the axial cup mass (^86^Sr) for 30s (20 cycles each 1.05 s), and secondly data collection involving a block of 50 cycles of 2 s integrated time. Interferences by Kr in the carrier gas (argon) and by Rb in both the carrier gas and samples were corrected, same as mass bias normalization (using ^88^Sr/^86^Sr=8.375209, exponential law), following an inverse mass bias correction procedure described by Nowell and collaborators [13].

A regression equation described by Copeland and collaborators [8] was used to estimate the strontium concentration (ppm) of the enamel solution runs, based on the ^88^Sr signal intensity (V) of three solutions with known strontium concentrations (100, 400 and 700 ppb). We used the strontium carbonate isotopic standard SRM 987 (NIST, USA) as working standard during the measurement, standard SRM 1486 as prepared external standard, and blanks as controls for contamination during the preparation. Thus, one analytical session was composed of 24 samples, 2 prepared blanks, 2 prepared standards SRM 1486, and 8 working standards SRM 987 with 16 blanks (one before and one after the working standard). Samples of this study were measured in two different analytical sessions.

Repeated ^87^Sr/^86^Sr measurements of working standard SRM_987 resulted in a mean of 0.710287 ± 0.000010 (1σ, n=16) during the analytical sessions and were corrected to the accepted value of 0.710240 ± 0.00004 [14,15]. The long-term average for ^87^Sr/^86^Sr of the external standard SRM 1486 is 0.709297 ± 0.000024 (n=68). The measurements of standard SRM 1486 resulted in a mean of 0.709297± 0.000011 (1σ, n=2) during the analytical sessions. All procedural blanks were considered negligible (^88^Sr < 0.040 V) at <0.4% of the analyte signal intensity (^88^Sr= ≈20V).

**Legends for the figures and tables**

**S1 Fig.** **Cranium of Burial 26.**

**S2 Fig.** **Frontal bone of Burial 26.** a) Picture of the right region of the frontal bone. The arrows point the incision; b); c) and d) SEM of the incision.

**S3 Fig.** **Confocal imaging of the incision located in the frontal bone (same as depicted in Figure 7)**. a) Three-dimensional model (above) and topography (bottom) based on the 20x lens (resolution of µm). The white dotted rectangle delimits the area shown in “b”; b) Three-dimensional model (above) and topography (bottom) based on the 50x lens (resolution of 1.57µm). Note how the incision has a flat bottom not compatible with a cut mark.

**S4 Fig. Right zygomaticof Burial 26.** Yellow arrows indicate the very thin incisions in the malar bone.

**S5 Fig. SEM and confocal microscopy of the incisions (green and white arrows) observed in the region of right zygomatic.**

**S6 Fig.**  **Right asterionic region of the cranium of Burial 26.** a) Picture of the posterior right portion of the cranium where incisions are present near the right asterion.

**S7 Fig. SEM of the right asterionic region of the cranium of Burial 26 (same as in figure S6)**. In low magnification (“a” and “b”) is possible to observe the sub-parallel orientation of the possible cut marks (indicated by the green arrows). In higher magnification some look more like v-shaped incisions (“c” and “d”) while others look more like broad striation (“e” and “f”).

**S8 Fig. Cervical vertebrae**. They were complete and well-preserved presenting no sign of fracture or breakage.

**S1 Table. Operation parameters for MC-ICP-MS solution analysis used at the Max-Planck Institute for Evolutionary Anthropology (Leipzig, Germany).**

**S2 Table. Craniometric variables used in this study.**

**S3 Table. Comparative series included in the craniometric analyses.**

**S4 Table. Classifications of Burial 26 according to Discriminant Function Analysis.**

**References of Supplementary Material**

1. Buikstra JE, Ubelaker D. Standards for data collection from human skeletal remains. Arkansas Archaeol Surv Res Ser. 1994;44.

2. Da-Gloria P. Health and lifestyles in the paleoamericans: early Holocene biocultural adaptation at Lagoa Santa. The Ohio State University. 2012.

3. Miles A. The dentition in the assessment of individual age in skeletal material. In: Brothwell D, editor. Dental Anthropology. Oxford: Pergamon Press; 1963. pp. 191–209.

4. Ubelaker D. Human skeletal remains. Washignton DC: Taraxacum Press; 1989.

5. Scott E. Dental wear scoring technique. Am J Phys Anthropol. 1979;51: 213–218.

6. Haglung W. Disappearence of soft tissue and the disarticulation of human remains from aqueous environments. J Forensic Sci. 1993;38: 806–815.

7. Kakarla UK, Chang SW, Theodore N, Sonntag VKH. Atlas fracture. Neurosurgery. 2010;66: A60–A66.

8. Copeland SR, Sponheimer M, Roux PJ, Grimes V, Lee-thorp JA, Ruiter DJ De, et al. Strontium isotope ratios ( 87 Sr / 86 Sr ) of tooth enamel : a comparison of solution and laser ablation multicollector inductively coupled plasma mass spectrometry methods. Rapid Commun Mass Spectrom. 2008;22: 3187–3194.

9. Deniel C, Pin C. Single-stage method for the simultaneous isolation of lead and strontium from silicate samples for isotopic measurements. Anal Chim Acta. 2001;426: 95–103.

10. Horwitz EP, Chiarizia R, Dietz ML. A Novel Strontium Selective Extraction Chromatographic Resin. Solvent Extr Ion Exch. 1992;10: 313–336.

11. Charlier BLA, Ginibre C, Morgan D, Nowell GM, Pearson DG, Davidson JP, et al. Methods for the microsampling and high-precision analysis of strontium and rubidium isotopes at single crystal scale for petrological and geochronological applications. Chem Geol. 2006;232: 114–133.

12. Batey JH, Prohaska T, Horstwood MSA, Nowell GM, Goenage-Infant H, Eiden GC. Mass Spectrometers. ICP Mass Spectrometry Handbook. Blackwell; 2005. pp. 26–116.

13. Nowell GM, Pearson DG, Ottley CJ, Schweiters J, Dowall D. Long-term performance characteristics of a plasma ionisation multi-collector mass spectrometer (PIMMS): the ThermoFinnigan Neptune. Plasma Source Mass Spectrom Spec Pub R Soc Chemestry. 2003; 307–320.

14. Terakado Y, Shimizu H, Masuda A. Nd and Sr isotopic variations in acidic rocks formed under a peculiar tectonic environment in Miocene Southwest Japan. Contrib to Mineral Petrol. 1988;99: 1–10.

15. Johnson CM, Lipman PW, Czamanske GK. H, O, Sr, Nd, and Pb isotope geochemistry of the Latir volcanic field and cogenetic intrusions, New Mexico, and relations between evolution of a continental magmatic center and modifications of the lithosphere. Contrib to Mineral Petrol. 1990;104: 99–124.
